# Aerodynamics Show Membrane-Winged Theropods Were a Poor Gliding Dead-end

**DOI:** 10.1016/j.isci.2020.101574

**Published:** 2020-10-22

**Authors:** T. Alexander Dececchi, Arindam Roy, Michael Pittman, Thomas G. Kaye, Xing Xu, Michael B. Habib, Hans C.E. Larsson, Xiaoli Wang, Xiaoting Zheng

**Affiliations:** 1 Department of Biology, Division of Natural Sciences, Mount Marty University, Yankton, SD, USA; 2 Vertebrate Palaeontology Laboratory, Division of Earth and Planetary Science, The University of Hong Kong, Hong Kong SAR, China; 3 Foundation for Scientific Advancement, Sierra Vista, AZ, USA; 4 Institute of Vertebrate Paleontology & Paleoanthropology, Chinese Academy of Sciences, Beijing, China; 5 Natural History Museum of Los Angeles County, Los Angeles, CA, USA; 6 Redpath Museum, McGill University, Montreal, QC, Canada; 7 Institute of Geology and Paleontology, Linyi University, Linyi City, Shandong, China; 8 Shandong Tianyu Museum of Nature, Pingyi, Shandong, China

**Keywords:** Paleontology, Biological Sciences, Evolutionary Biology, Paleobiology

## Abstract

The bizarre scansoriopterygid theropods *Yi* and *Ambopteryx* had skin stretched between elongate fingers that form a potential membranous wing. This wing is thought to have been used in aerial locomotion, but this has never been tested. Using laser-stimulated fluorescence imaging, we re-evaluate their anatomy and perform aerodynamic calculations covering flight potential, other wing-based behaviors, and gliding capabilities. We find that *Yi* and *Ambopteryx* were likely arboreal, highly unlikely to have any form of powered flight, and had significant deficiencies in flapping-based locomotion and limited gliding abilities. Our results show that Scansoriopterygidae are not models for the early evolution of bird flight, and their structurally distinct wings differed greatly from contemporaneous paravians, supporting multiple independent origins of flight. We propose that Scansoriopterygidae represents a unique but failed flight architecture of non-avialan theropods and that the evolutionary race to capture vertebrate aerial morphospace in the Middle to Late Jurassic was dynamic and complex.

## Highlights

• Flight potential of “membrane-winged” scansoriopterygian dinosaurs tested
• They had little ability to use flapping-based flight or locomotion
• Reduced gliding abilities compared with modern and fossil gliding animals
• Scansoriopterygians, a failed experiment in pre-bird theropod flight

## Graphical Abstract

**Figure:**
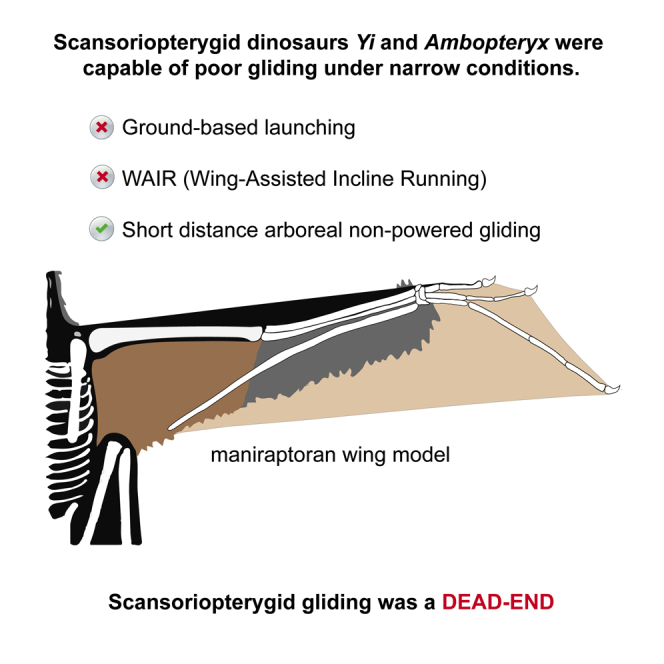

## Introduction

The origin of birds is one of the most studied vertebrate macroevolutionary transitions (Makovicky and Zanno, 2011; Zelenitsky et al., 2011; Brusatte et al., 2014, 2015; Xu et al., 2014b; Dececchi et al., 2016). The earliest avialans and their closest relatives possessed a relatively conservative body plan, differing little from the basic coelurosaurian condition (Brusatte et al., 2014). Overwritten upon this basic Bauplan are several overarching trends within theropods (Dececchi and Larsson, 2009; Makovicky and Zanno, 2011; Turner et al., 2012; Brusatte et al., 2014) such as body size reduction (Turner et al., 2007; Dececchi and Larsson, 2013; Lee et al., 2014; Benson et al., 2014), increased shoulder mobility (Turner et al., 2011), and large pennaceous feathers on the fore- and hindlimbs (Zheng et al., 2013; Xu et al., 2009, 2017; Lü and Brusatte, 2015; Li et al., 2012; Foth et al., 2014) that converge near the rise of avialans. Although the path to flight was complex, the relatively constrained nature of the stem avialan Bauplan, exemplified by the *Velociraptor*-type body form (*sensu* (Brusatte et al., 2014), has led to the assumption that this body plan applied to a single evolutionary trajectory to powered flight rather than multiple failed trajectories.

The discovery of *Yi qi* challenged this view. *Yi* is a small non-avialan theropod that possessed a unique combination of features speculated to have been used for gliding-like behaviors: reduced forelimb plumage and an extensive skin-based patagium supported by a “styliform element” (Xu et al., 2015; Larsson et al., 2020). *Yi* and *Ambopteryx,* a recently described taxon from coeval rocks (Wang et al., 2019), are members of the enigmatic Scansoriopterygidae (Pittman et al., 2020b), a clade of bizarre theropods with a hypertrophied fourth digit (Zhang et al., 2002) (herein we use the II-III-IV terminology for maniraptoran digit identity following embryological numbering and recent discoveries in earlier-diverging theropods; see (Xu et al., 2014b) for more details on the digit identity debate) and derived feather morphology (Zhang et al., 2008) whose phylogenetic position within Pennaraptora is uncertain (Xu et al., 2014b; Pittman et al., 2020a). The discovery of more than one membrane-winged and potentially volant taxon within this poorly known clade is intriguing, especially since other members show both arboreal and terrestrial adaptations (Zhang et al., 2002, 2008), implying high ecological diversity within this group. Their unique wing construction has implications for understanding the developmental plasticity of the theropod forelimb and evolutionary variation of theropods as they tested the stringent physical constraints of flight (Dudley et al., 2007; Xu et al., 2014b).

Using laser-stimulated fluorescence (Kaye et al., 2015; Falk et al., 2016; Wang et al., 2017; Serrano et al., 2020), we re-examine the type specimen of *Yi qi* (Xu et al., 2015), STM 31-2 (housed at the Shandong Tianyu Museum of Nature) to gain new insights into its skeletal and soft tissue morphology, chiefly in relation to the extent and shape of the patagium and the nature of the proposed styliform element. We reconstruct the most detailed forelimb anatomy of this bizarre taxon to quantitatively assess its speculated aerial capabilities. We apply these models to *Ambopteryx* to extend the ranges of estimated flight capabilities for the clade. Although the intralimb proportions differ between the two taxa, with *Ambopteryx* showing a more developed proximal limb region with the styliform representing only 32% of forelimb length (humerus + ulna + styliform element) as opposed to 42% in *Yi*, the total forelimbs are similarly elongated (both 4.65x femur length). Through a detailed morphological reconstruction, we seek to determine, for the first time, whether gliding or powered flight was possible for these taxa and whether a terrestrial or arboreal launch setting was required to achieve take-off for these flight modes. *Yi* and *Ambopteryx* are compared with the similarly sized paravians *Archaeopteryx* and *Microraptor* in terms of anatomy and flight potential (an appraisal is given in (Pei et al., 2020; Dececchi et al., 2020; Pittman et al., 2020c)). Using these new anatomical insights and their predicted flight potentials, we present a more thorough commentary on the broader evolutionary patterns of the origins of theropod flight. We present the relevant estimation methods such that these estimates can easily accommodate future discoveries and reconstructions.

## Results

### Extent of Soft Tissue Preservation

**Figure 1:**
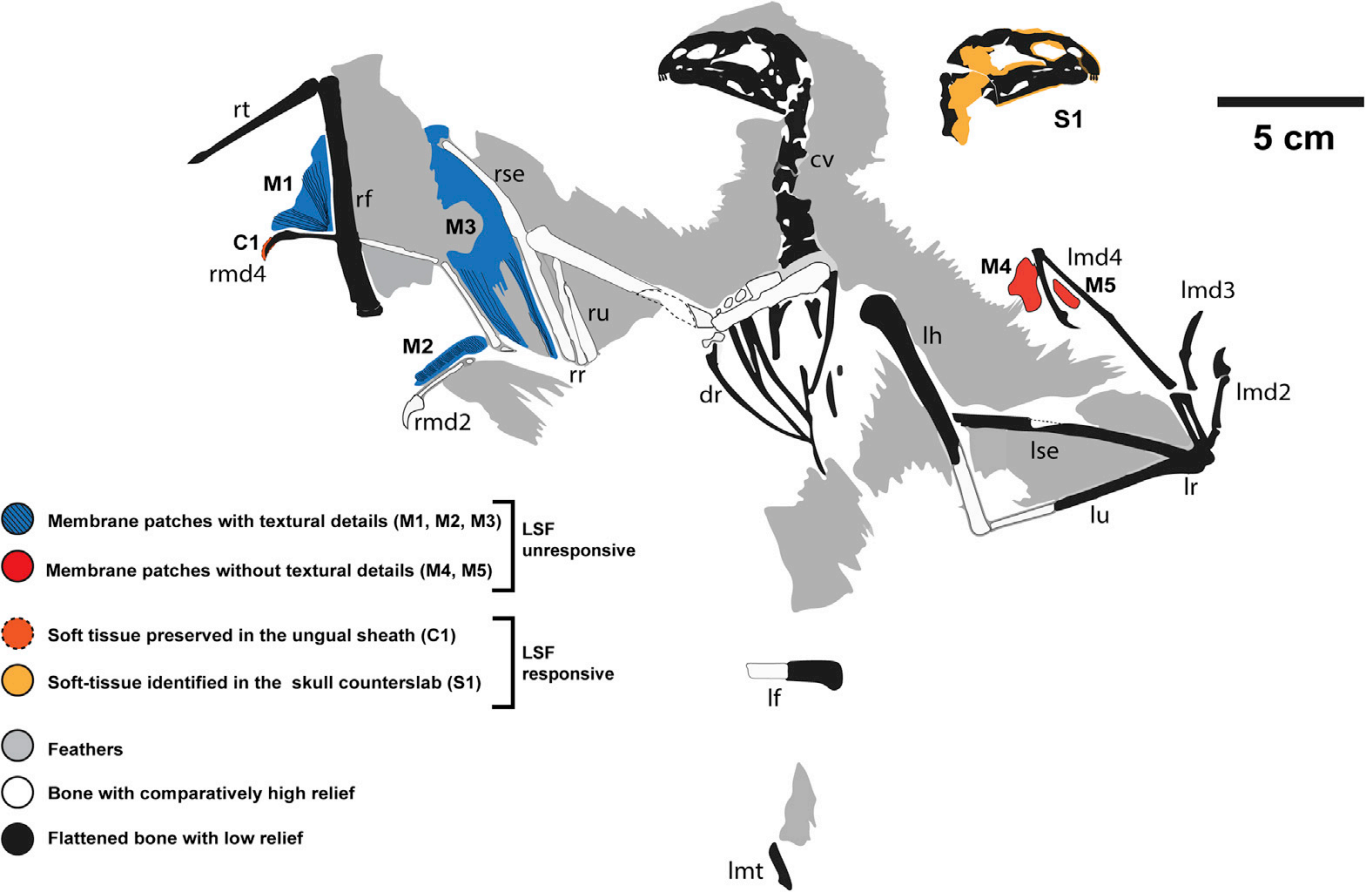
Soft-Tissue Map of *Yi qi* Map showing previously reported membrane patches by Xu et al. (2015) (M1–M5) as well as soft tissues identified with laser-stimulated fluorescence (LSF) (C1 and S1).

**Figure 2:**
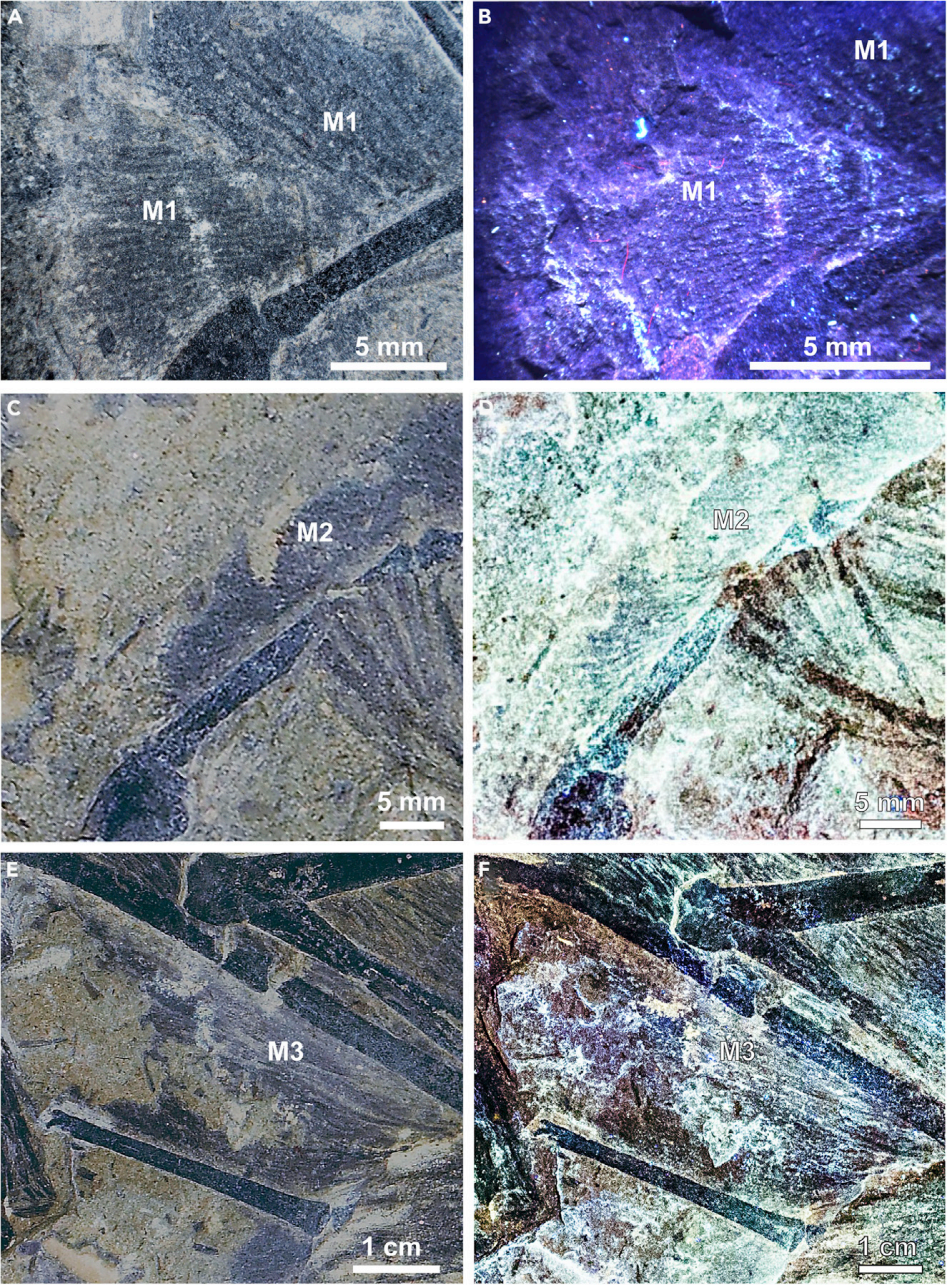
Parallel Striations in Membrane Patches 1, 2, and 3 Patch M1 next to right manual digit 4 (rmd4): (A) white light; (B) LSF. Patch M2 lying to the upper left of right manual digit 2 (rmd2): (C) white light; (D) negative LSF image. Patch M3 between right metacarpal IV (rmc) and right styliform element (rse): (E) white light; (F) LSF.

**Figure 3:**
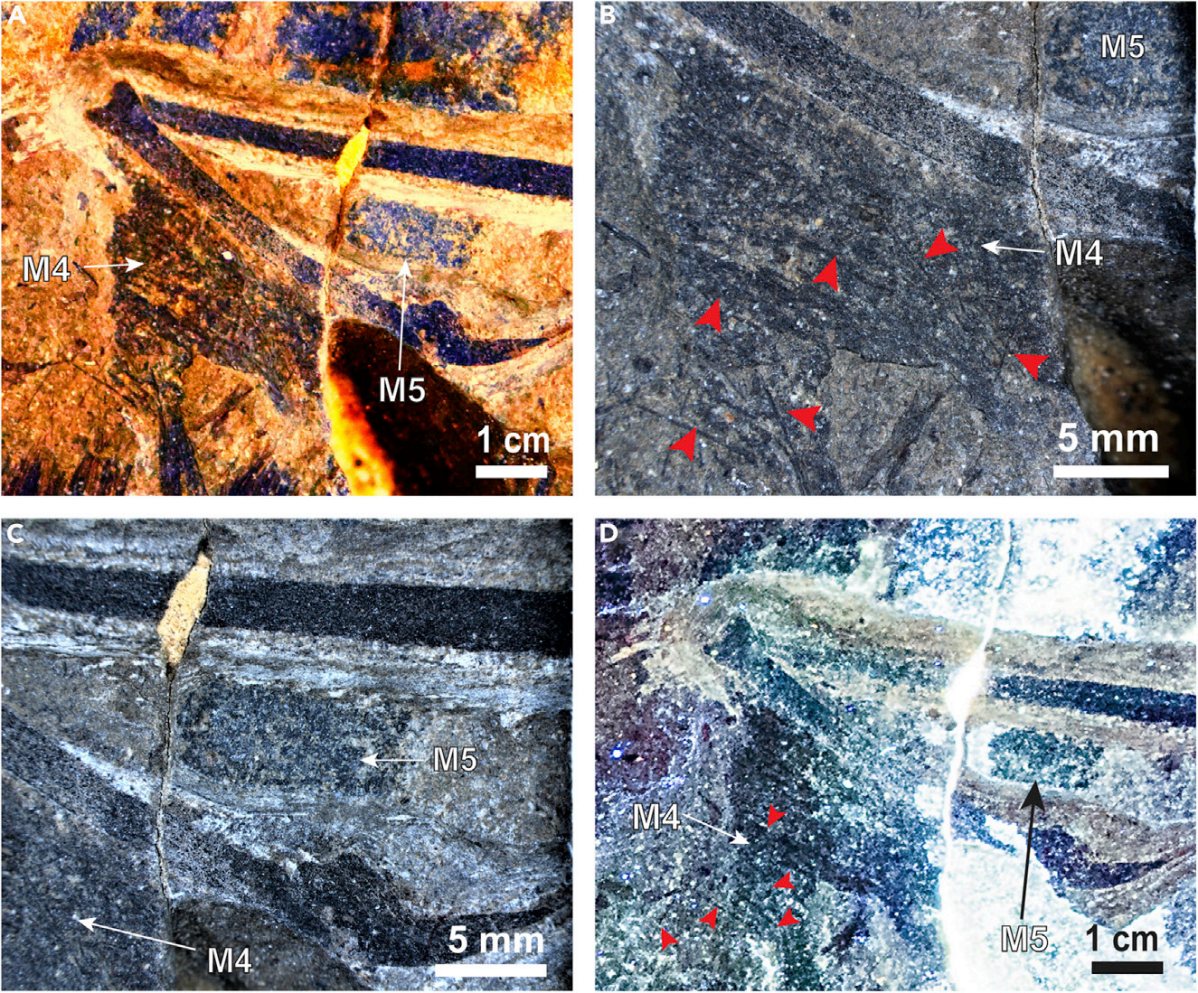
Membrane Patches 4 and 5, Left and Right of Left Manual Digit (lmd4) (A) Both patches M4 and M5 under white light (white arrows); (B) white light close-up of M4 showing its amorphous texture; (C) white light close-up of M5 showing the thin feather filaments passing through the membrane patch, giving it a false textured appearance; (D) LSF does not show any additional textural details otherwise hidden in white light images. Red arrowheads indicate feather filaments passing through M5.

**Figure 4:**
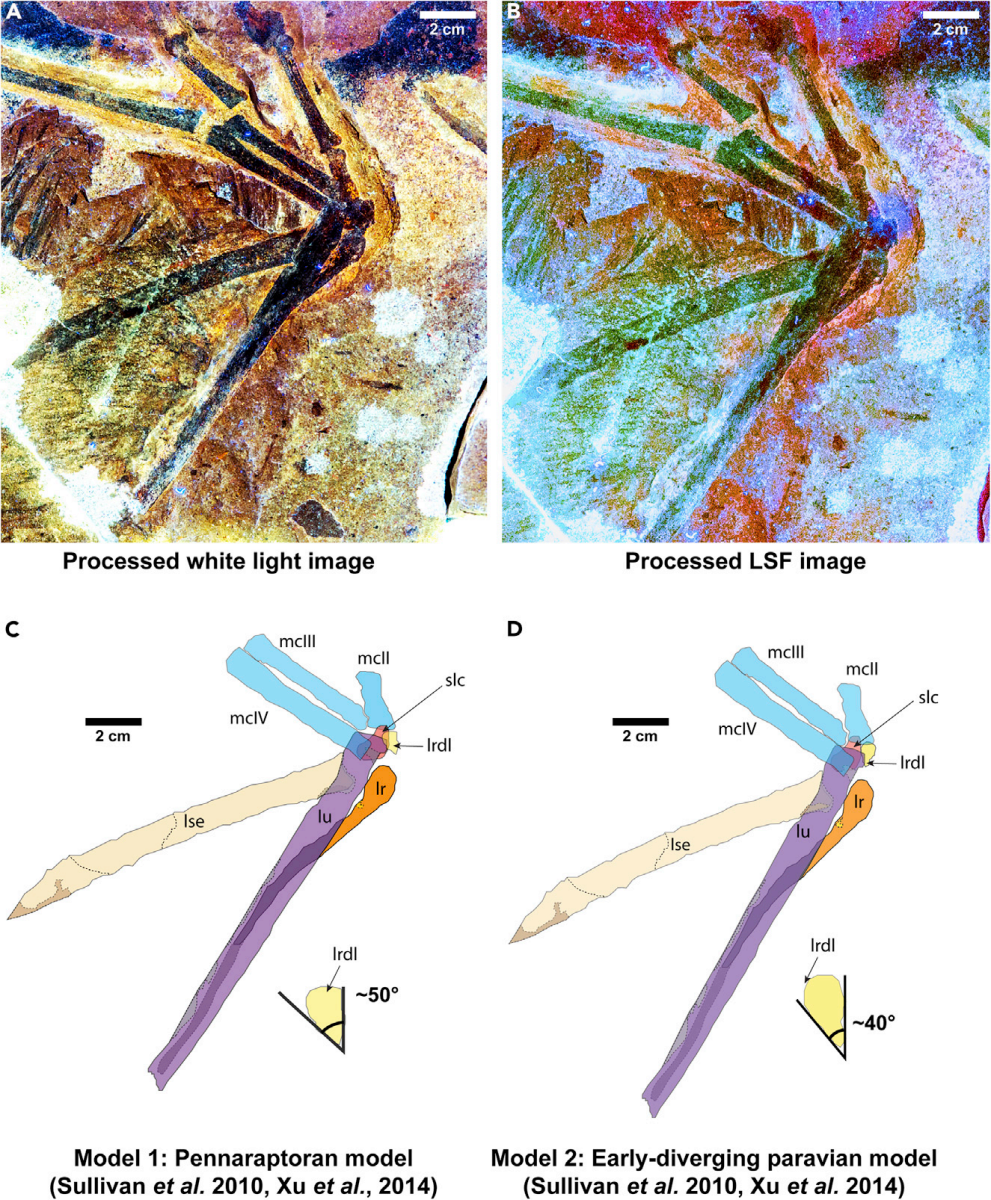
Reconstructed Anatomy of the Left Forelimb and Wrist of *Yi qi* (A) white light; (B) LSF; (C) radiale angle for a pennaraptoran model (model 1); (D) radiale angle for an early diverging paravian model (model 2). Scale: 2 cm.

STM 31-2 preserves filamentous feathers and skin patches on the slab and counterslab (Figures 1, 2, 3, and 4; Tables 1; S1).

**Table 1:** Soft Tissue Characteristics of *Yi qi*

Soft Tissue		Characteristics				Imaging Conditions
Reference Code	Location	Shape and Area	Low Relief/Flat Compared with Bones	Lighter in Color Compared with Nearby Bone and Feathers	Texture	
M1	Right manual digit 4 (rmd4)	Two triangular patches: lower (~1.05 cm^2^) upper (~2.33 cm^2^)	Yes, compared with rmd4	Yes	Parallel nonoverlapping striations (possibly collagen/elastin fibers) radiate at an angle to each other	Visible under white light and LSF
M2	Right manual digit 2 (rmd2)	Small rectangular patch (~1.62 cm^2^)	Yes, compared with rmd2	Yes	Parallel nonoverlapping striations (possibly collagen/elastin fibers), partly continuous in the counterslab	
M3	Between right metacarpal IV (rmc IV) and right styliform element (rse)	Large U-shaped patch (~10.33 cm^2^)	Yes, compared with rmc IV and rse	Gradation in color, right arm of the U-shaped lighter compared with left arm	Striations (collagen/elastin fibers) move from the base of the rse to and curve along with it to the apex. Overlaps with feather filaments	
M4	Left manual digit (lmd4) [left of]	Small tear-drop-shaped patch (~1.15 cm^2^)	Yes, compared with lmd4	Yes, lighter in color than lmd4, but very hard to distinguish color from feather filaments	Parallel striations as in 1 & 2 are found but not very clearly defined, fine feather filaments occur interspersed within and close to the patch	
M5	Left manual digit 4 (lmd4) [right of]	Small trapezoid patch (~0.37 cm^2^)	Yes, compared with lmd4	Yes, lightest color compared all patches	No striations observed	
C1	Claw sheath of rmd4	Curved around claw of rmd4	Yes	Faint outline under white light	–	Becomes much more prominent under LSF
S1	Skull	Multiple small patches occurring as halos on and around the bones of the skull—both upper and lower jaw, over premaxillary teeth, and postorbital (counterslab)	–	–	–	Only visible under LSF

#### Soft Tissues

Xu et al. (2015) proposed three criteria to characterize the patches of preserved membrane in *Yi qi*: (1) low relief and flatness, (2) lighter color than nearby bone or feathers, and (3) a striated texture presumed to be related to collagen/elastin fibers or taphonomic creasing of the skin. Xu et al. (2015) reported five patches (M1–M5) from the slab and one patch (S1) from the counterslab (see the numbering and location of the patches in Figure 1). Raking light microscopy clarified the texture in patches M1–M3 (Figure 2). Parallel striations as in patches M1, M2, and M3 are absent or not as clearly defined in patches M4 and M5, as morphotype 3 feather filaments (Xu et al., 2010) run across and clump in close proximity to the patches (see Figures 2 and 3). Soft tissue evidence was revealed under laser-stimulated fluorescence (LSF) in the skull region (Figure 1). These occur around the cranial bones in the counterslab, including around the upper and lower jaws, premaxillary teeth, around the edges of the orbit, and toward the posterior end of the skull but reveal no discernible anatomy. In addition, an ungual sheath (C1) was revealed for under LSF (Figure S1), but interestingly, the adjacent membrane patch M1 fluoresced negligibly.

### Wrist Folding and the Styliform Element

In the left forelimb, the radius overlays the ulna, whereas on the right the ulna overlaps the radius and the manus is supinated. In both cases the manus is supinated a full 180°, and the styliform element appears to remain on the ulnar side of the carpals (Figure 4). The distal ends of the radius and ulna show some raised topography, but the carpal bones are completely flattened. The wrist bones are slightly displaced from their natural configurations. Unfortunately, *Epidendrosaurus ninchengensis* (Zhang et al., 2002; Czerkas and A, 2002) and *Epidexipteryx hui* (Zhang et al., 2008) do not preserve fully articulated forearms preventing a full comparison. In *Ambopteryx* (Wang et al., 2019) the antebrachium is preserved but the carpal regions are either missing (left side) or poorly preserved (right side), making broader statements on the wrist morphology and mobility in this clade not possible.

Although the bones and teeth of *Yi qi* fluoresced negligibly under LSF, additional wrist morphology could be deduced because LSF caused the rock matrix to fluoresce, backlighting these bones (Figure 4). The distal radius has a well-defined hypertrophied condyle on its medial dorsal margin. This extensive condyle is absent in most theropods including birds, and the extension of this condyle onto the long axis of the radius suggests it may have facilitated a large degree of manual adduction. A clear semilunate carpal (SLC) is present and partially articulated to the proximal ends of metacarpals II, III, and IV (Figures 4C and 4D). The outline of the radiale can be inferred on the trochlear facet of the semilunate carpal (Figures 4C and 4D). Possible boundaries of other wrist bones are visible, but they cannot be confidently identified due to two embayed breaks on the distal head of the ulna. Interestingly, although the left hand has been supinated 180° from its natural position and the ulna displaced, the styliform element remains articulated to the carpus and underlies the ulna, suggesting the styliform element was tightly integrated into the carpus. A similar styliform element position can be inferred for the partial right manus. The integrated styliform element is reminiscent of the condition seen in flying squirrels (Thorington jr and Darrow, 2000).

**Figure 5:**
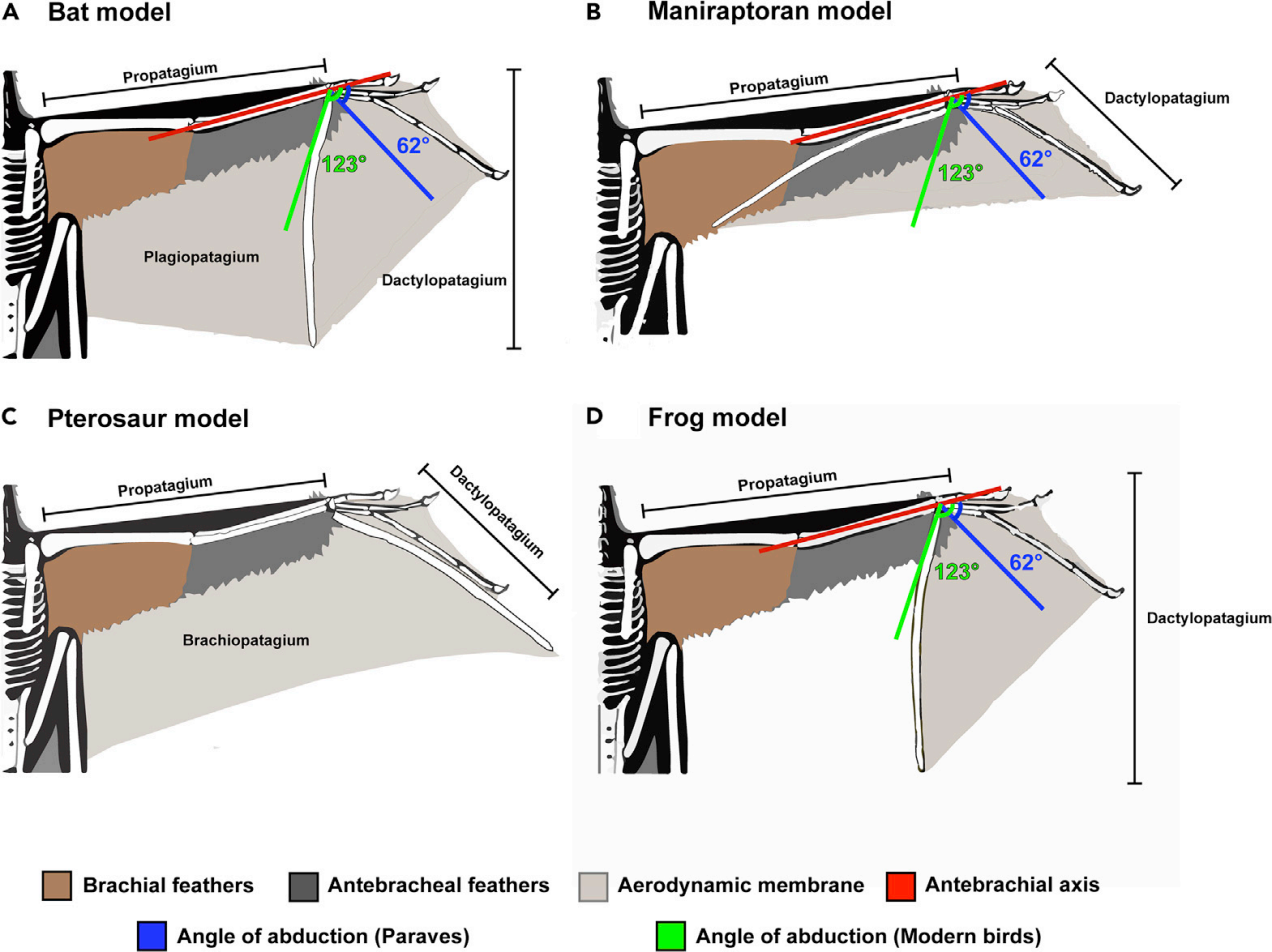
Revised Models of the Wing of *Yi qi* based on Xu et al., (2015) Dense stiff humeral feathers (dark brown), thinner forelimb feathers (dark gray), membranes (light gray), antebrachial axis (red), and two angles of abduction (~62° for paravians in blue and ~123° for modern birds in green) are shown. The ability to fold the wings toward the ulna is a trait that is thought to have a strong selection for in lineages leading to extant birds, although some earlier-diverging theropods may have also possessed this trait^29^. We should note that all these reconstructions show an extreme level of elbow extension, and this may be greater than in life. We follow the lead of Xu et al., (2015) in this respect and keep this uniform over all permutations.

Uncertainties in the morphology of the radiale and SLC lead us to propose two alternative models based on observations in other pennaraptorans (Sullivan et al., 2010; Xu et al., 2014a). The radiale angle, defined as the angle between the proximal face of the radiale and the facet articulating with the semilunar carpal (in non-avialan theropods) or carpometacarpus (in birds), is an osteological correlate that estimates the range of abduction (asymmetric folding of the avian hand toward the ulna) compared with adduction (folding of the avian hand toward the radius) (Sullivan et al., 2010). Larger radiale angles correspond to greater abduction rather than adduction. The radiale angle in the first “early diverging paravian model” is interpreted to be ~40° and for second “pennaraptoran model” it is ~50°, spanning the range of angles from Deinonychosauria (38°) to nodes Paraves (48°) and Pennaraptora (51°). These values are to be expected given that scansoriopterygids have been recovered as sister to oviraptorosaurians (Agnolin and Novas, 2013; Agnolin et al., 2019), earliest-diverging paravians (Godefroit et al., 2013) as well as early diverging avialans (Turner et al., 2012). This also means that *Yi* had a large angle of adduction (angle between an axis through the antebrachium and long axis through the metacarpus). The values would ideally be similar to those of *Deinonychus* (angle of abduction = 62°) and other paravians at one end (Senter, 2006) and modern birds (e.g. *Meleagris gallopavo*) on the other (angle of abduction = 123°) (Sullivan et al., 2010). However, it is to be noted that oviraptorosaurians show extremely large radiale angles (76°+) compared with non-avialan paravians, the early fossil bird *Eoconfuciusornis* (radiale angle = 55°), and modern birds (Sullivan et al., 2010). The range of folding may have been accordingly lessened in *Yi*, as the different positions and orientations of the styliform element would constrain wrist movement. Thus, if we assume that this folding is ideally maximized, it provides a means to evaluate the feasibility of the various wing models postulated in Xu et al. (2015) (see Figure 5, Table 1).

#### Wing Models of *Yi*

The tightly integrated styliform element to the wrist appears to support the feathered, membranous wing as proposed by Xu et al. (2015). Importantly, patch M3 shows the feather filaments attaching as a tight parallel sheet to the handward edge of the stylopodial element (Figure 2E). Neither stylopodial element preserves evidence of a membrane sheet attaching on the opposite edge, as a broad coat of feathers from the antibrachium and elbow cover this region. Moreover, the complete supinated rotation of both hands suggests the wing membrane was not extensively attached to the body flank nor hindlimb. In addition, the potential for a nearly 90° manual adduction could only be possible with a limited patagium extending proximally from the stylopodial element.

A range of wing models was assessed for their anatomical feasibility (Figure 5, Table S1). The models were named after the extent of the preserved aerodynamic membrane and their loose resemblance to bats, pterosaurs, maniraptorans, and flying frogs. The models are not strictly homologous to any of the structures in these aforementioned taxa. Although the stylopodial element may have been fixed in the position preserved in both hands, the maniraptoram model accounts for the full manual abduction capable for maniraptorans and is included to capture the widest range of possible wing architectures. In light of the data and analysis provided in this study, no single wing model of *Yi* satisfies the anatomical and functional factors examined here. Support for the maniraptoran model comes from phylogenetic conservation of anatomy, including carpal angles and a lack of evidence of a plagiopatagium. Although both the bat and the pterosaur models would increase wing area, thus reducing loading values and increasing gliding capabilities, both lack any direct evidence of the hindlimb attachment sites. In addition, if the styliform element is fixed to the carpus, the complete 180° supination of the manus would be less likely if it connected with a patagium bound extensively to the body and hindlimb. The frog model is considered the least likely. Although it is supported by the lack of evidence of a plagiopatagium or a branchiopatagium and the fact the styliform element appears tightly integrated to the carpus, it would provide a vastly smaller wing area, thus increasing wing loading values (especially at mid to larger mass estimates) well beyond the upper bounds of flight capacity. The lack of evidence for a corresponding hindfoot “wing” would decrease stability and limit mobility during descent. It is also not supported by the findings of feathered structures supported by something resembling a patagium in *Ambopteryx* (Wang et al., 2019) and *Epidendrosaurus* (Czerkas and Feduccia, 2014). Thus, the two best-fitting models are maniraptoran and bat (Table S1). In each of these the angular orientation of the styliform element with respect to metacarpal IV would not have allowed for easy rotational flapping of the wings, hence making a strong case for a gliding/arboreal lifestyle.

### Was Powered Flight Possible?

**Figure 6:**
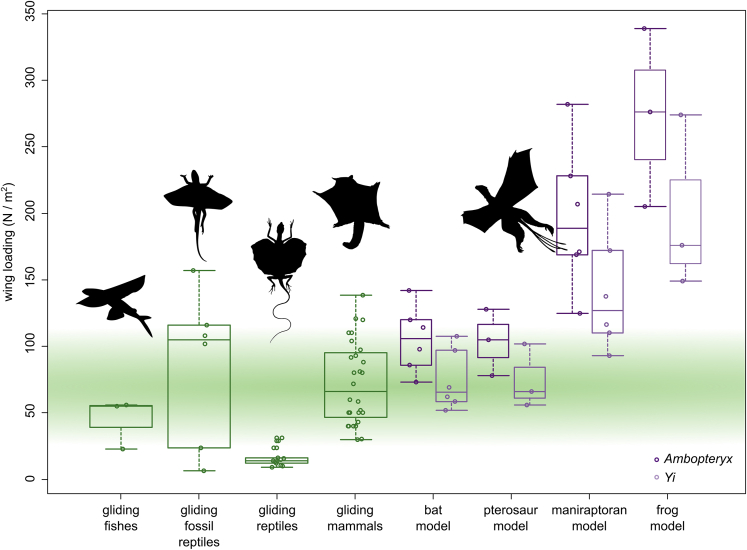
Comparison of Wing Loading in Extant and Extinct Gliders with Four Different Wing Models of *Yi* and *Ambopteryx* Boxplots are derived from wing loading values for gliders and for different wing shapes and mass estimates of *Yi* and *Ambopteryx* presented in Tables S2 and S3. Note that in most cases loading values for scansoriopterygids are less than the 245 Nm^−2^ maximal value for allowing flight (Meunier, 1951). The green ban spans most of the wing loading values recorded for gliding mammals. Silhouettes are original or from Phylopic.org and used under a Creative Commons Attribution 4.0 International License (CC BY 4.0).

All body mass permutations, except *Ambopteryx* under the highest mass and lowest wing area estimation, recover wing loadings below 245 Nm^−2^, suggesting some form of flight was possible in *Yi* and *Ambopteryx* (Table S2). Powered flight requires extensive pectoral musculoskeletal adaptations to support powerful flight musculature. No evidence of these are present in scansoriopterygids, leaving gliding as the only possibility of flight for these taxa. Wing loadings for both scansoriopterygids under a bat and pterosaur wing model were comfortably within the range seen in extant and extinct gliders (Figure 6). However, the maniraptoran and frog models lie well outside known glider wing loadings. The specific lift criteria are more equivocal. At the highest mass estimate, *Yi* did not obtain lift values sufficient to achieve take-off although it is possible at lower masses (body sizes that have been previously challenged as too light (Wang et al., 2019)) (Table S4). Only at the highest power output and lowest body mass estimates for *Ambopteryx* was take-off achievable. If we decrease flight muscle mass to 8% of total mass (as estimated for other non-paravian pennaraptorans (Allen et al., 2013)) it precludes powered take-off except at the highest muscle power output levels (see (Dececchi et al., 2016) for further discussion). This lower pectoral mass estimate is suggested by the lack of sterna coupled with the relatively small deltopectoral crests in these taxa (Xu et al., 2015; Wang et al., 2019). In *Yi* the deltopectoral crest is only 19% of total humeral length and 22% in *Ambopteryx*, both of which are much smaller values than those seen in *Microraptor* (29%) or *Archaeopteryx* (34%). In *Microraptor* or *Archaeopteryx,* 14 of 15 permutations adopting 10% flight muscle mass and 12 of 15 permutations adopting 8% flight muscle mass achieve sufficient lift values. Combining these two criteria indicates borderline powered flight potential for *Yi* and less so for *Ambopteryx* (Dececchi et al., 2020; Pei et al., 2020).

Even if we assumed powered flight was achievable, we also examined the power curves for each taxon using a pectoral mass fraction of 10% and various drag coefficients (see Methods) using the software program *Flight 1.24* (Pennycuick, 2008). By comparing power availability (Templin, 2000) to minimum mechanical power required we can see if a taxon could achieve and sustain flapping flight, assuming launch is successful. With the exception of the lowest mass estimated for both *Yi* and *Ambopteryx*, we find that insufficient power is generated under realistic drag scenarios for flight (coefficient of drag (Cd) 0.4 and 0.5 based on Serrano et al., 2020) under both the maniraptoran-forewing-only (smallest wing area) and bat fore + hindwing models (largest wing area) (Table 2). In contrast, the comparable sized *Archaeopteryx* and *Microraptor* specimens succeed under all Cd permutations. Although it is suspected that larger birds have lower Cd values approaching 0.2 (Hedenstrom and Liechti, 2001), this still results in minimal power requirements above what could be produced for the larger *Yi* mass estimate (0.7kg). As power availability is estimated based on extant birds and given that we assumed a generous flight muscle fraction of 10% for these values, it is likely that power availability would be even lower in scansoriopterygids than calculated here, making powered flapping flight even less probable.

**Table 2:** Powered Flight Comparison between *Yi*, *Ambopteryx*, and Selected Paravian Specimens

Taxon	Model	Mass (kg)	Pwr	Min Cd0.1	Min Cd0.4	Min Cd0.5
*Yi qi*	MFW	0.38	5.24	3.41	4.82	5.09
	BBW	0.38	5.24	3.93	5.56	5.88
	MFW	0.45	5.94	4.52	6.40	6.77
	BBW	0.45	5.94	6.93	9.81	10.40
	MFW	0.7	8.23	9.43	13.40	14.10
	BBW	0.7	8.23	14.50	20.50	21.70
*Archaeopteryx*	Berlin	0.2	3.26	1.59	2.24	2.37
*Microraptor gui*	BMNHC PH881	0.18	3.01	1.35	1.91	2.02
	BMNHC PH881	0.24	3.73	2.19	3.09	3.27
	IVPP V13352	0.95	10.32	8.45	12.00	12.60
	IVPP V13352	0.5	6.42	3.61	5.10	5.39
*Ambopteryx*	MFW	0.23	3.61	2.88	4.07	4.30
	BBW	0.23	3.61	4.35	6.16	6.51
	MFW	0.31	4.51	4.73	6.69	7.08
	BBW	0.31	4.51	7.16	10.10	10.70
	MFW	0.38	5.24	6.65	9.40	9.94
	BBW	0.38	5.24	10.10	14.20	15.00

Pwr denotes available power based on Templin (2000), Min Cd denotes minimum mechanical power required for flight under one of the three coefficients of drag permutations (0.1, 0.4, 0.5). See text for details. MFW = maniraptoran/avian wing style construction forewing area only, BBW = bat wing style construction both fore and hindwing area.

### Terrestrial Based Take-Off

Assuming powered flight was possible, it seems unlikely that *Yi* could achieve it from a ground-based take-off scenario and impossible barring extremely liberal assumptions for *Ambopteryx*. In *Yi*, only under the single permutation of a “bat wing area” model with a bird flapping frequency is a minimum take-off speed achievable through running. Other permutations show minimum take-off speeds between 1.1 and 2.9 times the maximum possible sprint speed (Table S5). In *Ambopteryx* the minimum take-off speed is 2.3–3.9 times top sprint speed. In no case with or without flapping assistance was leaping sufficient to achieve launch. This is in sharp contrast to *Microraptor* or *Archaeopteryx* that are estimated to have achieved take-off speeds across all permutations (Table S5).

As the previous results only examine running without adding wing thrust, we included the effect of flap-assisted running on take-off (Burgers and Chiappe, 1999). Using this method, we see only 2 permutations of *Yi*—the 380g and 450g models using the bat wing area configuration with bird wingbeat frequency—that achieve sufficient lift to support their body weight and none for *Ambopteryx* (Table S6). Where it occurs, it takes between 5 and 6 s after starting the run, slower than seen in *Microraptor* (less than 2 to 6 s) or *Archaeopteryx* (between 2 and 3 s). Higher flap angles, which are likely not possible for scansoriopterygids but available to paravians, do not significantly alter this (see Supporting Information). Lowering the coefficient of lift (Cl) to either 1.5 or 1 has a significant effect in increasing the time it takes for take-off. At a Cl of 1.5, *Yi* does not achieve take-off until after 7 s of sprinting (see Supporting Information) and not at all under a Cl of 1. This suggests that ground-based take-off may have been restricted to taxa, which had a modified shoulder girdle providing a greater stroke angle, a feature that appears to be restricted to Paraves and absent in the Scansoriopterygidae.

Another form of flapping based locomotion, wing-assisted incline running (WAIR), may be possible in *Yi* (Table S7) but contingent on which model is chosen. Using an avian flapping model, sufficient force is generated to allow for the possibility of level I WAIR across body size estimates, although at a significantly lower level than seen in comparable paravians. Under a bat flapping frequency, WAIR capability is significantly curtailed, especially at high masses. *Ambopteryx* only achieves the lowest WAIR cut-off under the bat area model with bird flapping.

### Gliding

Extant gliding taxa tend to have smaller maximum body masses and lower wing loading values than powered flyers (Tables S3 and S8): maximum body mass between 2.5 and 3 kg (Dial, 2003) and wing loading values in extant gliders ranging from 9–143 Nm^−2^. The Triassic fossil amniote *Kuehneosaurus* possibly extends the range of the latter to ~157 Nm^−2^, though the gliding ability of this taxon has been questioned (Stein et al., 2008; McGuire and Dudley, 2011). *Yi* and *Ambopteryx* are both within these ranges, suggesting gliding flight was possible assuming a bat and pterosaur wing model (Figure 6), except at the highest mass estimate for *Yi*. However, high wing loadings suggest *Yi* and *Ambopteryx* may not have been effective gliders, as estimated glide speeds are high but not beyond what is seen in extant taxa (Motokazu and Shiraishi, 1993; Byrnes et al., 2008; Chatterjee and Templin, 2007). Recently Amador and colleagues (Amador et al., 2019) demonstrated a significant gap in aspect ratio and wing loading between bats and gliding mammals. We find that the membrane-winged non-avialan theropods, although remaining within the avian morphospace, trend more toward gliders compared with *Microraptor*, *Archaeopteryx,* and extant powered fliers (Table S8).

Compared with other proposed gliding or flying theropods (Yalden, 1971; Ostrom, 1974; Burgers and Chiappe, 1999; Chatterjee and Templin, 2003, 2007; Xu et al., 2003; Alexander et al., 2010; Dyke et al., 2013), both scansoriopterygids show poorly developed gliding abilities. At similar body sizes both *Yi* and *Ambopteryx* show significantly higher glide speed, sinking speed, and circle radius (set at 24° bank in *Flight 1.24* (Pennycuick, 2008)) than either *Microraptor* or *Archaeopteryx* (Table 3). This indicates that gliding flight in scansoriopterygids would require higher speeds, higher launch points, and be less precise than other paravians.

**Table 3:** Gliding Flight Comparison between *Yi*, *Ambopteryx*, and Selected Paravian Specimens

Taxon	Model	Mass (kg)	Coefficient of Drag 0.4				Coefficient of Drag 0.5			
			Glide ms^-1^	Sink ms^-1^	Glide ratio	Radius	Glide ms^-1^	Sink ms^-1^	Glide ratio	Radius
*Yi qi*	MFW	0.38	10.70	0.81	13.30	21.70	10.30	0.85	12.10	21.70
	BBW	0.38	9.80	1.02	9.58	11.90	9.40	1.04	9.03	11.90
	MFW	0.45	11.30	0.89	12.70	26.10	11.20	0.98	11.50	26.10
	BBW	0.45	10.40	1.12	9.30	14.10	10.00	1.11	8.74	14.10
	MFW	0.7	13.90	1.26	11.10	42.40	13.30	1.34	9.90	42.40
	BBW	0.7	12.30	1.44	8.56	21.70	11.70	1.46	8.00	21.70
*Archaeopteryx*	Berlin	0.2	8.00	0.69	11.60	9.01	7.70	0.70	11.00	8.72
*Microraptor gui*	BMNHC PH881	0.18	8.00	0.67	12.00	9.95	7.60	0.68	11.20	9.33
	BMNHC PH881	0.24	8.90	0.79	11.30	12.60	8.50	0.80	10.60	11.60
	IVPP V13352	0.95	10.80	0.84	12.80	19.00	10.30	0.86	11.90	19.00
	IVPP V13352	0.5	8.90	0.68	13.10	12.30	8.50	0.69	12.30	11.60
*Ambopteryx*	MFW	0.23	11.70	1.22	9.59	33.10	11.60	1.34	8.66	33.10
	BBW	0.23	10.70	1.30	8.24	17.70	10.20	1.33	7.67	16.90
	MFW	0.31	13.60	1.55	8.75	45.70	13.60	1.74	7.80	45.70
	BBW	0.31	11.90	1.54	7.73	21.10	11.30	1.58	7.17	20.80
	MFW	0.38	15.10	1.85	8.17	56.40	15.10	2.09	7.22	56.40
	BBW	0.38	12.80	1.73	7.39	26.60	12.20	1.78	6.84	24.10

Glide denotes best glide speed in ms^−1^, sink denotes sink speed in ms^−1^, radius denotes banking radius at 24° in m. MFW = maniraptoran/avian wing style construction forewing area only, BBW = bat wing style construction both fore and hindwing area.

## Discussion

### Extent of Soft Tissue Preservation

Enhanced white light and LSF images do not reveal the parallel striations reported by Xu et al. (2015) but do image subparallel feather filaments running across them (Figure 3). These orientations suggest the wing membranes were covered with closely spaced, elongate feather filaments, reminiscent of the feather-like integument structures recently found in pterosaurs (Yang et al., 2019). The finger claw sheath makes the claws more recurved and suggests an arboreal lifestyle unlike what is seen in *Epidexipteryx* (Zhang et al., 2008). However, all previously reported membranes (M1–M5) show a negligible response to LSF (Figures 1, 2, and 3). Either pervasive mineral replacement has obscured some organic tissues, the membranes are covered with tightly spaced feather filaments, or perhaps the patches are not membranes at all. This latter suggestion is unrealistic given the presence of the styliform element and purported membranous structures in *Ambopteryx* (Wang et al., 2019) and *Epidendrosaurus* (Czerkas and Feduccia, 2014). Our findings suggest a more restricted patagial extent than originally modeled (Xu et al., 2015) (see Supporting Information). This finding is congruent with the identification of propatagia in oviraptorosaurians (Feduccia and Czerkas, 2015), early branching paravians (Wang et al., 2017), and early avialans (Zheng et al., 2017) bracketing the range of possible phylogenetic positions for scansoriopterygids, and all showing a more typical maniraptoran type forelimb/wing. Thus, the maniraptoran model, which is more closely allied with the early diverging paravian wing plan (e.g. that of *Anchiornis*), should be prioritized in comparisons in light of this imaging data (Figure 5). If future specimens show that the patagium was even more restricted indicating a smaller lifting surface (e.g. similar to *Anchiornis* (Wang et al., 2017)), the values chosen here should be considered as upper bounds. In this case, it would indicate even more restrictive aerial behavioral repertories than those reported here.

### Implications for Scansoriopterygid Paleoecology

The behavioral repertoire and phylogenetic position of Scansoriopterygidae have been areas of debate (Zhang et al., 2008; Brusatte et al., 2014; Xu et al., 2014b, 2015) and have important implications for our understanding of the diversity of pre-avialan theropods. However, relatively fragmentary preservation and juvenile ages for most specimens make it difficult to make clear statements on locomotory aspects for this group (Dececchi and Larsson, 2011). *Yi* and *Ambopteryx* specimens strengthen the original assessments of the potential scansorial or arboreal life history of at least some members of this clade (Zhang et al., 2002; Czerkas and A, 2002; Dececchi and Larsson, 2011). The seemingly terrestrial nature of *Epidexipteryx* is suggested by its reduced feathering, elongate curial index, and low claw curvature (Zhang et al., 2008) and is nesting among terrestrial theropods in paleoecological analyses (Dececchi and Larsson, 2011). Given that, it is possible that the patagium-based wing of *Yi* and *Ambopteryx* had an aerodynamic function while having evolved in a terrestrial context, a scenario also suggested for the Jurassic mammal *Volaticotherium* (Meng et al., 2017).

We find these taxa show little ability to utilize their elongated forelimbs to aid locomotion in a terrestrial setting. The inability of *Yi* and especially *Ambopteryx* to even approach take-off potential (except under the most extreme permutations) is indicative of the limited likelihood for this taxon to take to the air if grounded. This is in stark contrast to *Archaeopteryx* or *Microraptor*. Although the ability to perform limited WAIR does seem plausible, the values generated are low such that slight modifications to any parameter, such as reducing speed to those seen in juvenile chukars (Tobalske and Dial, 2007), significantly reduces the potential for this behavior. In addition, the flight musculature of Scansoriopterygidae was reduced, as it had an underdeveloped deltopectoral crest, unlike *Microraptor* or *Archaeopteryx* (Hwang et al., 2002; Pei et al., 2014), and a small sterna (Wang et al., 2019), unlike the condition seen in *Microraptor* (Xu et al., 2003). This supports a reduced power output compared with *M. gui* or *Archaeopteryx*. When factored into the reconstructions of powered flight potential, especially those of short duration that are more physiologically and behaviorally more likely (Erickson et al., 2009; Dececchi et al., 2016) but more energetically costly (Nudds and Bryant, 2000), it makes take-off and sustained flight (even for a short duration) practically impossible for *Yi* and *Ambopteryx* except at the most generous settings. It should also be noted that these muscle output values for take-off are based on modern quick burst flight specialists (Tobalske and Dial, 2000) and not expected to occur in a transitional flier. This combined with the lack of consistent achievement of minimum power requirements, even in assuming modern avian-like parameters that are highly unlikely in this non-paravian with reduced flight muscle capacity and size, further argues against flapping flight appearing in these scansoriopterygids.

This leads to the suggestion that if the patagium of *Yi* and *Ambopteryx* does have some locomotory usage (Xu et al., 2015), it was used for nonpowered gliding flight. Our wing loading estimates vary but fall within the range seen in extant and extinct gliders (<143Nkg^−1^), assuming a bat and pterosaur wing model (Figure 6, Tables S2 and S3). No single-wing model is fully supported by the anatomical and wing loading results, suggesting the wings of scansoriopterygids may have been a hybrid between several models. In particular, a mixed bat and maniraptoran wing model may best align with known anatomy and required wing loading of a glider. McGuire and Dudley (2011) discussed how higher loading values reduce performance values (Table 3) suggesting scansoriopterygids were poor gliders. In addition, the wings of *Yi* and *Ambopteryx* are primarily composed of rigid bony elements, thus reducing its flexibility and manoeuvrability with no evidence of specialized muscles or other supporting elements, such as elastin within the patagium that controls wing shape in bats (Cheney et al., 2017), gliding mammals (Socha et al., 2015), and pterosaurs (Kellner et al., 2010; Palmer, 2018; Yang et al., 2019). With limited flexibility and aeroelastic properties of the patagium, the high glide speeds estimated here that are often associated with lower manoeuvrability hint that although gliding was possible it was likely more similar to that suspected in other fossil reptilian gliders (Li et al., 2007; Stein et al., 2008; McGuire and Dudley, 2011) than in many extant mammalian ones. In equilibrium gliding, higher wing loading and higher glide speeds lead to increased height loss per distance traveled and limits aerial manoeuvrability (Table 3) (see (Dudley et al., 2007) and references therein).

Nonequilibrium gliding as would be expected during short-duration glides (Bahlman et al., 2013; Socha et al., 2015) creates a more complicated model, but the high wing loading values estimated here suggest relatively large amounts of height loss per horizontal distance traveled and high glide speeds (Dyke et al., 2013; Socha et al., 2015). The values found here would likely mean that gliding would not be an energetically efficient means of movement through their forest habitat. With such highly loaded wings and high speeds of movement, the total costs of climbing to a sufficient launch point to achieve a desired horizontal distance would likely be quite energetically expensive (Byrnes et al., 2011). This may explain why not all members of this clade, notably *Epidexipteryx*, appear to have adopted this lifestyle (Zhang et al., 2008; Dececchi and Larsson, 2011). The lack of clear energetic benefits raises the questions as to what the drivers for gliding would be in this lineage.

### Patagium and Its Implications for Avian and Flight Origins

A patagium-based flight apparatus has repeatedly evolved across different vertebrate clades (Dudley et al., 2007; Socha et al., 2015). Although the drivers to enter the aerial realm are likely nonuniform, multifaceted, and reliant on the evolution history of each lineage (Socha et al., 2015), some overarching patterns are apparent. The first is that, for all nonaquatic gliders, an arboreal life history is linked to gliding flight with a suspicion that the origin of an extended skin-based gilding surface is related to having gone through a parachuting phase (Essner, 2002; Dudley et al., 2007). This is then accentuated in advanced gliding or powered flying forms with either the repurposing of existing muscles or development of new ones to help gain a greater control over the flight surface (Thorington jr and Darrow, 2000; Thorington jr and Santana, 2007; Tokita et al., 2012). The mere presence of a patagium itself is not indicative of flight capabilities. One is purportedly present in the early branching oviraptorosaurian *Caudipteryx* (Feduccia and Czerkas, 2015) whose body size and diminutive wings generate wing loading values that indicate it is was not capable of flight (Dececchi et al., 2016; Pei et al., 2020).

The discovery of *Yi* and *Ambopteryx* with their unique patagium is the most direct evidence of arboreality within non-avialan theropods. Their lack of ability to utilize this structure in a terrestrial setting, the presence of a styliform element (Xu et al., 2015; Wang et al., 2019), enlarged phalangeal index (PI) of *Yi* (manual digit III = 1.7, digit IV = 4.35), and higher levels of claw curvature than seen in more terrestrial clade members such as *Epidexipteryx*, all suggest a tree canopy dwelling lifestyle. Similar traits are present in other Scansoriopterygidae even if they do not preserve a patagium and styliform element. Although juvenile, specimens of *Epidendrosaurus* have a high pedal PI, a classic signal for arboreality (Zhang et al., 2002), although their early ontogeny advises caution. As the hindlimb responds to selection in arboreal taxa quicker than the forelimb (Rose, 1987; Meng et al., 2006), we would expect to see changes in this region earlier. Building on the work of Dececchi and Larsson (2011) and using the pattern of changes we see in both *Yi* and the more complete *Epidendrosaurus*, we can reconstruct the series of changes to the theropod Bauplan we expect to see to make non-avialan theropods into efficient arboreal climbers and gliders. The low curial index (tibia/femur) of *Epidendrosaurus* (1.17) compared with *Epidexipteryx* (1.25) does not appear to be linked to ontogeny because it does not significantly shift with growth in other theropods such as oviraptorids, *Microraptor* or *Archaeopteryx* (Table S9), and the low value is consistent with climbers (Dececchi and Larsson, 2011). Although *Ambopteryx* shows relatively elongated distal limbs, this may be because the femur is reduced in this taxon (Wang et al., 2019) as is its entire hindlimb compared with terrestrial maniraptorans (Tables S10 and S11). In addition, the position of Mt I in *Epidendrosaurus* is such that the trochleae of Mt I-IV are nearly level with an elongated digit I (Zhang et al., 2002) creating a more stable grasping surface than in other non-avialan theropods. If we reconstruct *Yi* with similar arboreal features in addition to the patagium, we see a body form unlike that in other pennaraptorans, suggesting a significantly different life history.

### Conclusions

The findings presented here, including the discovery of integumentary features and the presence of feathers lining the purported patagium, permits a detailed anatomical and aerodynamic analysis that informs our inferences of other members of this still poorly known clade. We find that the most likely reconstruction for *Yi* and *Ambopteryx* follows a mixed bat and maniraptoran model, indicating that the membrane may have been an adapted and exaggerated expression of the pro- and postpatagium found in other theropods (Kaye et al., 2015; Falk et al., 2016; Wang et al., 2017). We infer the styliform element to be relatively fixed to the posterior edge of the carpus and that the membranous, feathered patagium associated with the manus articulates along its handward edge.

Aerodynamic modeling suggests that *Yi* and *Ambopteryx* were obligate gliding organisms, likely to be arboreal, with gliding abilities similar to those seen in similar-sized mammalian gliders, both extant and extinct. They show significant differences and deficiencies in flapping-based locomotion such as WAIR or ground-based launching compared with similar-sized fossil paravians who deployed a more “typical” avian flight apparatus (*Archaeopteryx*, *Microraptor*). Unlike previous assertions of scansoriopterygids representing models for early bird flight evolution, we propose that this clade was an independent colonization of the aerial realm for non-avialan theropods. If true, this would represent at least two, but more likely three or more attempts at flight (both powered and gliding) by small pennaraptoran theropods during the Mesozoic (See Pei, Pittman et al., 2020). Given the large number of independent occurrences of gliding flight within crown mammals, this should perhaps be unsurprising, but it does create a more complex picture of the aerial ecosystem. Currently there are no definitive scansoriopterygids outside the latest Middle-earliest Late Jurassic Yanliao biota. This implies a short duration for this lineage suggesting its radiation was short lived. The presence of potential competitors or predators in the Tiaojishan Formation (and coeval beds) such as different lineages of mammalian gliders (Meng et al., 2006, 2017; Luo et al., 2017) and pterosaurs (Sullivan et al., 2014) perhaps restricted the available ecospace. Birds became flight capable sometime in the mid to late Jurassic with more competent take-off capabilities very early in their history and gained access to the arboreal realm soon after (Dececchi and Larsson, 2011). The relatively poor gliding abilities of *Yi* and *Ambopteryx* coupled with their inability to achieve take-off unless at high starting point elevations would have been a significant disadvantage compared with these other aerial vertebrates with more manoeuvrable and capable flight styles. Our results suggest that scansoriopterygid maniraptorans might have been specialists in a particular type of forest habitat structure, perhaps those with frequent, small gaps. Their relatively high glide speeds and average glide ratios would have been suitable for quickly crossing small gaps in the canopy, whereas these would have made longer flights less efficient and exposed them to higher predation risk from the more accomplished contemporaneous flying pterosaurs. Although speculative, the idea that they were habitat specialists of some nature would explain both their limited glide performance and relatively short duration in the fossil record. Scansoriopterygids therefore appear to have been a failed experimental lineage of early arboreal gliders unable to compete with the rapidly evolving mammalian gliders and avialan fliers of the time.

### Limitations of the Study

Although our study does account for air density estimates, body mass permutations, and wing shape permutations, we have no information on the wing cross-section in *Yi qi* or its relatives. As a result, we have assumed that the aerofoil section was similar to that of living-membrane-based flyers and that the wings of *Yi qi* could generate maximum lift coefficients commensurate with such a section. This relatively liberal estimate may overestimate flight performance for *Yi qi*, particularly with regard to manoeuvrability. Furthermore, we also assume that *Yi qi* was capable of enough active membrane control to reach similar maximum lift to drag ratios as other membrane flyers. The wings of living bats and extinct pterosaurs, in particular, are/were sophisticated and capable of exceptional active surface control. It is quite possible that *Yi qi* had a much more simple membrane structure that lacked such control, making our maximum efficiency estimates quite generous. Finally, although we consider the issue of proper wing membrane tensioning by only considering wing shapes that could tension the membrane, we did not make an attempt to reconstruct the material properties of the wing membrane itself or determine the likely aeroelastic limits in a quantitative fashion. It is possible that this would further confine the potential range of wing shapes.

### Resource Availability

#### Lead Contact

Further information, requests, and inquiries should be directed to and will be fulfilled by the Lead Contact, T. Alexander Dececchi (alex.dececchi@mountmarty.edu)

#### Materials Availability

This study did not generate new specimens or materials. All images are included in the text and Supporting Information.

#### Data and Code Availability

The published article includes all data generated or analyzed during this study.

## Methods

All methods can be found in the accompanying Transparent Methods supplemental file.

## Supplemental Information

Document S1. Transparent Methods, Figure S1, and Tables S1–S4

Table S5. (A) Minimum Take-off Velocity Compared to Maximum Running and Leaping Speed and (B) Body Weight Support Values at Maximum Running Speed, Related to Main Text and Transparent Methods (A) Minimum estimated take-off speed under flap running and jumping compared to estimated maximum running speed at Froude number of 15. Note that in only one case - using a bat wing area model, the lowest mass value and the highest bird flap range - is *Yi* capable of achieving minimal take-off speed. (B) Body weight support generated at maximum running speed for different mass and wing area models for *Yi*, *Microraptor* and *Archaeopteryx*. Note that for the later paravian taxa two different flap amplitude estimates were used to correspond to their ability to generate a larger amplitude flight stroke. Amp= flap amplitude in m, Bat= bat flapping frequency in Hz, Bird= bird flapping frequency in Hz, Cl= coefficient of lift, Flap=flap angle in radians, Mass is in g. Speed is in ms^-1^.

Table S7. WAIR Support Values at Reduced Body Speeds and Flap Angles, Related to Main Text and Transparent Methods (A) 70° flap angle, speed 1.2 ms-1, (B) 50° flap angle, speed 1.2 ms-1, C) 70° flap angle, speed 0.6 ms-1.

Table S8. Comparison of Aspect Ratio versus Wing Loadings in Birds, Bats and Non-avian Theropods, Related to Main Text and Transparent Methods Data on wing loading and aspect ratio in modern bats and mammalian gliders from Amador *et al*. 2019 updated to included avian powered fliers, *Yi*, *Microraptor* and *Archaeopteryx*. Avian data from Greenwalt 1975.

Table S9. Hindlimb Measurements for Theropods, All Measurements in mm, Related to Main Text and Transparent Methods Allo=allosauroids, Alv= Alvarezsauroids, Anchi=anchiornithids, Archae=*Archaeopteryx*, Basal= basal theropods, Cera=ceratosauroids, Com=compsognathids, Coel= basal coleurosaurs, Drom=dromaeosaurids, Ovi= oviraptorosaurs, Ornitho=ornithomimosaurs, Tyran=tyranosauroids, F=femur length, T=tibial length, Mt=metatarsal length.

Table S10. Relative Limb Length against either Snout to Vent Length (SVL) or Trunk (Tr) Length across Non-avian and Basal Avian Theropods, Related to Main Text and Transparent Methods All measurements in mm.

Table S11. Regression for Hindlimb Length (mm) versus Femoral Circumference in Theropods, Related to Main Text and Transparent Methods (A) Hindlimb length (femur +tibia + metatarsus) length regressed against the femoral circumference for both OLS (log limbL= 0.78663log Fcir (+/- 0.020042) + 1.3668 (+/- 0 0.04017), r= 0.95654, p<0.0001) and RMA OLS (log limbL = 0.80431log Fcir (+/- 0.020042) + 1.3323 (+/- 0 0.0016134), r=0.95654, p<0.0001). Rank denotes the relative position of the residual compared to other taxa with the lower number denoting a taxon with a longer metatarsus than expected. All measurements in mm. (B) *Yi* and *Ambopteryx* expected versus reconstructed hindlimb values and where they would rank amongst non-avialan theropods. For *Yi* we ran two different femoral circumferences and hindlimb length permutations. Using the equations in Wang et al. (2019) we get a femoral diameter estimate of ~6.1mm, slightly larger than we obtain from measuring the images (5.7mm). To account for this, we ran both. As for hindlimb, Xu et al. (2015) reconstructed *Yi*’s hindlimb proportion based on *Epidexipteryx*. If *Yi* is proportioned more similarly to *Ambopteryx*, due to them both being arboreal and *Epidexipteryx* being terrestrial, this would alter estimated hindlimb length. Therefore, we ran both reconstructions for each femoral circumference estimate.

## Footnotes

Supplemental Information can be found online at https://doi.org/10.1016/j.isci.2020.101574.
