# Supplementary material for: Aerodynamics Show Membrane-Winged Theropods Were a Poor Gliding Dead-end
Source: iScience. 2020 Oct 22;23(12):101574. doi: 10.1016/j.isci.2020.101574 (PMC7756141; doi:10.1016/j.isci.2020.101574)
Supplement: Document S1. Transparent Methods, Figure S1, and Tables S1–S4 [file mmc1.pdf]

iScience, Volume *23*

Supplemental Information

Aerodynamics Show Membrane-Winged

Theropods Were a Poor Gliding Dead-end

T. Alexander Dececchi, Arindam Roy, Michael Pittman, Thomas G. Kaye, Xing Xu, Michael B. Habib, Hans C.E. Larsson, Xiaoli Wang, and Xiaoting Zheng

Transparent Methods

Laser-Stimulated Fluorescence (LSF)
Yi qi was imaged using LSF following a modified version of the protocol of Kaye *et al*. 2015 (see (Wang, Pittman et al. 2017)). This involved capturing the fluorescence produced by the specimen during long exposure photos when a 0.6 W 405 nm laser was raster-scanned over its surface. Images were equalised and colour balanced in *Photoshop CS6*.

Aerodynamic analysis
Detailed equation-based aerodynamic calculations of terrestrial-based running launch take-off and flight capabilities for *Yi* and *Ambopteryx* were made using the new details provided by LSF augmented imaging. These results were compared to known vertebrate flyers to draw the study's main conclusions.
Terrestrial based running and launch take-off
We examined several different methods for calculating the minimal threshold this taxon would have to overcome through either of these two methods to achieve a successful launch. The first is to determine what is the minimal speed required to have achieve lift values more than body weight. This was generated using the methodology of Dececchi *et al*. (2016) by rearranging the equation for lift production:
bw=0.5Cl*p *(fAmp +U)2 S/9.8*M
Body weight (bw) was set equal to 1, which denotes the amount of lift force needed to equal the downward force of gravity and is thus the minimal lift force needed to permit take-off. The partial pressure of air (p) was set to 1.23, flapping frequency was based on the "modified value" regression from (Dececchi, Larsson et al. 2016) and the coefficient of lift was 1.5. U is speed in ms^{-1}, M is mass in kg and FAmp is flap amplitude which is the wing length in m times the flap angle in radians. We ran two permutations for flap angle, at 70° (1.22 radians) or 50° (0.87 radians) to account for different expectations of maximal humeral elevation. As described in Dececchi et al. (2016) , this value for the coefficient of lift was selected as it approaches that seen during extant bird take-off (Usherwood 2009).

The resulting minimal velocity was then compared to those generated either through a running start, a static leap or a wing assisted leap. For running a maximum value was calculated using estimated hip height of 80% total hindlimb length (femur +tibia + Mt) which corresponds to the upright leg seen in cursorial birds such as ostriches (Birn-Jeffery, Hubicki et al. 2014). Froude value, a dimensionless number representing the flow dynamics of an object in a medium, is used -amongst other things- in the study of running dynamics in animals [(Vaughan and O'Malley 2005)], was set at 15. This indicates a very fast sprint but is similar to the maximum estimated for other theropods and within the range seen in modern running birds (Cottam, Williams et al. 1942, Hutchinson and Garcia 2002, Sellers and Manning 2007, Dececchi, Mloszewska et al. 2020). For leaping take-off, both wing-assisted and without, we used the equations found in (Dececchi, Larsson et al. 2016) with wing musculature set at 10% total mass and hindlimb+caudofemoralis musculature set at 30% total mass. These values are similar to those estimated for paravians in (Allen, Bates et al. 2013). We examined take-off velocities at 90° angles as it gave the highest values and assigned wing beat frequency and CI as the same as when we calculated minimal take-off velocity. We further investigated the ability for taxa to achieve sufficient lift and thrust using a wing-assisted running methodology by following the procedures and values from (Burgers and Chiappe 1999) over a 10 second time frame. If a specimen could achieve lift values

that equalled their body mass, they were deemed capable of take-off through this method. As the estimated coefficient of lift used in (Burgers and Chiappe 1999) of 2 is significantly higher than that seen in modern bird take-off (1.64 per (Usherwood 2009)), we also ran two permutations using lower Cl values of 1 and 1.5 that more closely resemble those seen during both take-off and WAIR in modern birds (Tobalske and Dial 2007, Heers, Tobalske et al. 2011) .

Finally, we examine the possibility of wing-assisted incline running in *Yi* using the methodology from (Dececchi, Larsson et al. 2016) . The shoulder girdle of Scansoriopterygidae does not show an extension of the glenoid onto the external surface of the scapula, which is associated with a lateral facing glenoid, nor other characters associated with elevation of the humerus above the level of the scapula (Zhang, Zhou et al. 2008, Turner, Makovicky et al. 2012). This coupled with the extreme elongation of the wing, its length is here reconstructed at 1.4x the total hindlimb length and 1.8x estimated hip height, suggests a complete downstroke in a terrestrial or inclined setting (such as with WAIR) was not possible. Thus, we chose to use 50° and 70° flap angles across *Yi*, *Ambopteryx*, *Microraptor* and *Archaeopteryx* as these more accurately encompassed the likely range of limb motion possible during WAIR, if it was present, that is common in all taxa. For wing beat frequency we chose the modified regression of (Dececchi, Larsson et al. 2016) as it included all birds in the dataset and gave the highest possible flapping frequency. We also selected a coefficient of lift (=1) and body speed (1.5 ms^{-1}) based on those suggested in (Dececchi, Larsson et al. 2016). These represent upper limits of likely values and thus set an upper threshold of performance outputs.

Gliding

In calculating possible glide speeds, we chose to us two different methodologies to gain a range of values of potential glide speeds. First, we modified the glide speed estimated equation from(Alexander, Gong et al. 2010) :

$mg/S= 0.38vg^2$

Where g= acceleration due to gravity, m= mass, S= wing area and vg is glide velocity. This reconstruction was selected as it allowed for an estimation of glide speed that did not require *a priori* knowledge of the coefficient of lift. We also chose to follow the methodology of (Stein, Palmer et al. 2008) for other Mesozoic gliding taxa:

$Vg=(2mg/pSCl)^{1/2}$

Where p is the density of air (1.23 kgm^{-3}) and Cl is the coefficient of lift. Recent work has documented long term atmospheric changes in air density across the Mesozoic and commented on how it could alter flight capacity (Serrano, Chiappe et al. 2019). We have incorporated thise as a separate permutation using the values from (Serrano, Chiappe et al. 2019). For *Yi* and *Ambopteryx* this means air density was set to the estimated value 160 MYA (1.171 kgm^{-3}). For *Archaeopteryx* this was set to the estimated value 150 MYA (1.178 kgm^{-3}) whilst for *Microraptor* this was set to the estimated value 120 MYA (1.209 kgm^{-3}). By running both these permutations, we can compare flight potential across taxa using both an assumption of common atmospheric conditions as well as those estimated when they lived. To account for the uncertainty in this last variable we ran two permutations for Cl of 1 and 1.5. This is based on the range seen in other estimates for extinct gliders and encompass the upper range seen in extant gliders (Stein, Palmer et al. 2008, Dyke, De Kat et al. 2013).

References

Alexander, D. E., E. Gong, L. D. Martin, D. A. Burnham and A. R. Falk (2010). "Model tests of gliding with different hindwing configurations in the four-winged dromaeosaurid Microraptor gui." Proceedings of the National Academy of Sciences **107**(7): 2972-2976.

Allen, V., K. T. Bates, Z. Li and J. R. Hutchinson (2013). "Linking the evolution of body shape and locomotor biomechanics in bird-line archosaurs." Nature **497**(7447): 104.

Birn-Jeffery, A. V., C. M. Hubicki, Y. Blum, D. Renjewski, J. W. Hurst and M. A. Daley (2014). "Don't break a leg: running birds from quail to ostrich prioritise leg safety and economy on uneven terrain." Journal of Experimental Biology **217**(21): 3786-3796.

Burgers, P. and L. M. Chiappe (1999). "The wing of Archaeopteryx as a primary thrust generator." Nature **399**(6731): 60.

Cottam, C., C. S. Williams and C. A. Sooter (1942). "Flight and running speeds of birds." The Wilson Bulletin **54**(2): 121-131.

Dececchi, T. A., H. C. Larsson and M. B. Habib (2016). "The wings before the bird: an evaluation of flapping-based locomotory hypotheses in bird antecedents." PeerJ **4**: e2159.

Dececchi, T. A., A. M. Mloszewska, T. R. Holtz, Jr., M. B. Habib and H. C. E. Larsson (2020). "The fast and the frugal: Divergent locomotory strategies drive limb lengthening in theropod dinosaurs." PLOS ONE **15**(5): e0223698.

Dyke, G., R. De Kat, C. Palmer, J. Van Der Kindere, D. Naish and B. Ganapathisubramani (2013). "Aerodynamic performance of the feathered dinosaur Microraptor and the evolution of feathered flight." Nature Communications **4**: 2489.

Heers, A. M., B. Tobalske and K. P. Dial (2011). "Ontogeny of lift and drag production in ground birds." The Journal of Experimental Biology **214**: 717-725.

Hutchinson, J. R. and M. Garcia (2002). "Tyrannosaurus was not a fast runner." Nature **415**(6875): 1018.

Sellers, W. I. and P. L. Manning (2007). "Estimating dinosaur maximum running speeds using evolutionary robotics." Proc. R. Soc. B **274**: 2711-2716.

Serrano, F. J., L. M. Chiappe, P. Palmqvist, B. Figueirido, J. Long and J. L. Sanz (2019). "The effect of long-term atmospheric changes on the macroevolution of birds." Gondwana Research **65**: 86-96.

Stein, K., C. Palmer, P. G. Gill and M. J. Benton (2008). "The aerodynamics of the British late Triassic Kuehneosauridae." Palaeontology **51**(4): 967-981.

Tobalske, B. and K. P. Dial (2007). "Aerodynamics of wing-assisted incline running in birds." The Journal of Experimental Biology **210**: 1742-1751.

Turner, A. H., P. J. Makovicky and M. A. Norell (2012). "A review of dromaeosaurid systematics and paravian phylogeny." Bulletin of the American Museum of Natural History **371**: 1-206.

Usherwood, J. R. (2009). "The aerodynamic forces and pressure distribution of a revolving pigeon wing." Experiments in fluids **46**(5): 991-1003.

Vaughan, C. L. and M. J. O'Malley (2005). "Froude and the contribution of navel architecture to our understading of bipedal locomotion." Gait and Posture **21**: 350-362.

Wang, X., M. Pittman, X. Zheng, T. G. Kaye, A. R. Falk, S. A. Hartman and X. Xu (2017). "Basal paravian functional anatomy illuminated by high-detail body outline." Nature Communications **8**: 14576.

Zhang, F., Z. Zhou, X. Xu, X. Wang and C. Sullivan (2008). "A bizarre Jurassic maniraptoran from China with elongate ribbon-like feathers." Nature **455**(7216): 1105.

Supporting Figure 1 related to Figure 1

Ungual sheath on right manual digit 4. Orange fluorescing ungual sheath (C1) is next to membrane patch 1 (big white arrow) which was unresponsive to LSF.

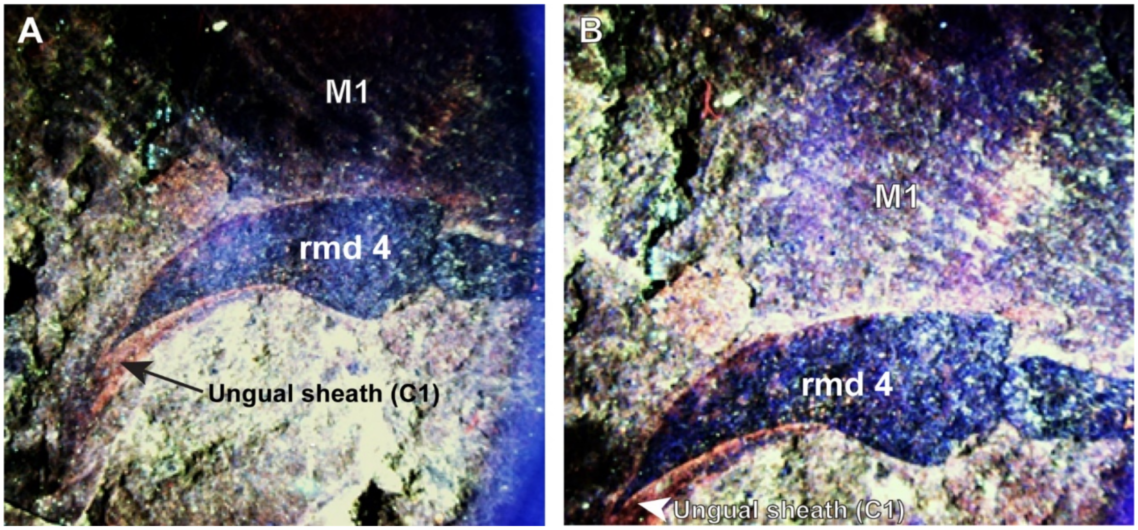

Supporting Table captions

Supporting Table 1. Related to figure 5

Summary of the wing models of *Yi qi* and their validity considering new data. Relative support for the purposed wing models of *Yi qi*: maniraptoran model > bat model > pterosaur model > frog model.

Model	Model summary	Revised fit of model
Bat	*Assumptions*[1]<br>- Styliform element positioned on the ulnar side, pointing medially.<br>- Largest aerodynamic surface lateral to trunk. Inclusive of an inferred propatagia, dactylopatagia within the digits. Two large sections: one bound by digit IV & styliform element and the other by styliform element & trunk.<br>- Feathers have been postulated to be present in dorsal and/or ventral surface.<br><br>*Traits*<br>- Largest surface area.<br>- Least phylogenetically conservative model<br><br>*Weaknesses*<br>- No membrane preserved posterior to the humerus and ulna.<br>- Length and high density of feathers along the humerus and ulna adds to drag.<br>- Low aspect ratio wing.	a) Styliform element positioned at an acute angle to MC IV (Fig. 7 a-c in Extended Data of [1]). This allows abduction of the hand at the paravian angle of abduction (~62°) but falls short of the angle in modern birds (~123°).<br>b) Long filamentous feathers along the antebrachium may have affected aerodynamic stability negatively.<br>c) The large membrane surface is useful to bats due to a two-fold control mechanism: (1) the collagen/elastin fibre network in the wing membranes are able to adjust camber in a dynamic way to various flight conditions[2]; (2) the highly articulated phalangeal bones allow morphing of the effective area of the wing and deformation along the plane of the wing[3]. Since the styliform element of *Yi* is one rigid bone and its soft tissue details are currently inconclusive, it is unlikely such maneuverability and camber control was possible, despite a large membrane surface.<br>d) Bat species with contrasting feeding ecologies demonstrate that species feeding primarily on non-mobile food (e.g. fruit) have fewer fully active joints than species that catch mobile prey (e.g. insects). It is hypothesized that there is a functional trade-off between energetic savings and maneuverability[4]. Having fewer joints and muscles reduces the mass of the wing, thereby reducing the energetic requirements of flapping flight. Having more joints increases the assortment of possible 3D wing shapes, thereby enhancing the range and fine control of aerodynamic force production and thus maneuverability. The reduced number of carpal bones and joints in *Yi qi* therefore suggests that it had more restricted control of aerodynamic force production.<br><br>**Score: 3/5**
Pterosaur	*Assumptions* [1]<br>- Styliform element positioned on the ulnar side, follows the curvature of MC IV rather than being oriented medially.<br>- Large aerodynamic surface lateral to trunk. Inclusive of an inferred propatagia, brachiopatagia and dactylopatagia.<br>- Two large sections of the aerodynamic membrane - bounded by MC IV and styliform element and brachiopatagium between styliform element and trunk.<br>- Feathers on dorsal/ventral surface.<br>- Relatively high aspect ratio wing compared to the bat model.<br><br>*Traits*<br>- Large surface area.<br>- High aspect ratio wing.<br><br>*Weaknesses*<br>- High density and length of feathers along the humerus and ulna adds to drag.	a) The pterosaur model assumes lateral rotation of the styliform element such that it follows the curvature of MC IV (Supplementary Data and Figs. 7 a-c in Extended Data of [1]). The angle between the styliform element and MC IV is <10°, which makes it highly restrictive to the abduction. Does not allow any folding of the hand towards the ulna.<br>b) A large membrane surface posterior to the styliform element. Actinofibril-like striations are present in membrane patches 1, 2 and 5. Similar striations are also routinely observed in the membranous wings of pterosaurs, which show that the upper actinofibril layer lies on muscular fascia and a vascular system [5,6]. However, the weak responsiveness of the membranes to LSF precludes visualisation of the inner layers and the texture could possibly have come from the uppermost epidermal layer.<br>c) None of the patches occur beyond the posterior margin of the humerus and the ulna. Poor support for this model from extent of preserved membranes.<br>d) Long filamentous feathers along the antebrachium and dense clumps of feathers make have increased

	- No membrane preserved posterior to the humerus and ulna.	drag to dangerous levels, potentially affecting aerodynamic stability.<br><br>**Score: 2.5/5**
Maniraptoran	*Assumptions* [1]<br>- Styliform element positioned on the ulnar side, directed towards the torso.<br>- Moderately large membrane surface (smaller than bat and pterosaur models). Includes inferred propatagium and presence of dactylopatagium.<br>- Membrane takes up the function of vaned flight feathers up to proximal end of the ulna. Vaned feathers replaced by thin feather filaments.<br>- Stiff feather filaments along the posterior margin of the humerus and ulna.<br>- Combines membrane and feather surface to produce a high aspect ratio wing.<br><br>*Traits*<br>- The composite wing (membrane + feathered posterior margin of forelimb) has a high aspect ratio.<br>- Consistent with evidence of preserved membranes: no membrane present posterior to the humerus or ulna.<br>- Most phylogenetically conservative model.<br><br>*Weaknesses*<br>- Higher wing loading compared to bat model.<br>- Stiff overlapping humeral feathers (even though not vaned) can create an airfoil, but extent of overlap is uncertain.	a) Reconstructed semi-lunate carpal closely resembles the paravian condition.<br>b) Radiale angle for both the pennaraptoran model and basal paravian model ranges from ~40-50°, which also lie between the ancestral state values corresponding to the different proposed phylogenetic placements of Scansoriopterygidae (as a non-paravian sister group to oviraptorosaurs[7], basal most paravians[8,9], and as basal avialans [10]).<br>c) For this model the position of the styliform element at an obtuse angle to MC IV allows the maximum possible abduction of the hand towards the ulna, and can accommodate both the paravian angle of abduction (~62°) and the modern bird one (~123°).<br>d) Consistent with extent of the preserved soft tissue patches, which do not go beyond the posterior end of the forelimb.<br>e) High-aspect ratio composite wing with stiff filamentous feathers probably produced a coherent sheet-like airfoil, but humeral feathers usually play a more minor role in modern bird flight. Whether the sparse, long and thin antebrachial feather filaments can adequately supplement the membrane-based airfoil is an open question and ids the main caveat to this model.<br><br>**Score: 4/5**
Frog	*Assumptions* [1]<br>- Styliform element positioned on the ulnar side, directed towards the medially.<br>- Assumes a dactylopatagium between the fingers with the section between MC IV and the styliform element being the principal aerodynamically functional portion.<br>- The plagiopatagium is absent, replaced by the stiff filamentous feathers running along the posterior margins of the humerus and ulna.<br><br>*Traits*<br>- The combined membranous and feathered wing has a relatively high aspect ratio, a point of resemblance to the maniraptoran model.<br>- Consistent with the positions and extent of preserved membranes.<br><br>*Weaknesses*<br>- Least membrane surface area of all proposed models.<br>- The major membrane surface is placed distal to the wing, thus causing high wing loading.<br>- Rhacophorid frogs glide using membrane surfaces on both the fore- and hind limbs.	a) Assumes the smallest extent of the membranes, with the only major membrane surface being between MC IV and the styliform element.<br>b) The filamentous feathers running along the posterior margins of the forelimb, in the absence of plagiopatagium on one hand would not produce sufficient airfoil and on the other have the disadvantage of high levels of drag.<br>c) Position of the styliform element at an acute angle to MC IV (Supplementary Data and Figs. 7 a-c in Extended Data of [1]). This allows abduction of the hand at angle found in paravians (~62°), but falls slightly short of modern bird (~123°).<br>d) The small membrane surface may not be sufficient to balance the weight of the body posterior to the forelimbs: unlike rhacophorid frogs, *Yi* does not have a membrane- supported hindlimb.<br><br>**Score: 2/5**

Supporting Table 2. Related to Figure 6.

A) Mass and wing permutations for *Yi*, *Ambopteryx* and comparable paravians using the Maniraptoran and Bat models. B)Using the pterosaur and frog models . Due to the low support (see Supporting Table 1) we do not suggest these models as likely representations for the wing design of *Yi* or *Ambopteryx*. They are included here for completeness only

A)

Taxon	Wing area (m^2)	Wing length (m)	Span (m)	Mass (kg)	Wing loading (Nm^{-2})	Notes
Yi qi STM 31-2	0.032	0.286	0.6	0.38	116	Maniraptoran wing area (forewing only)
	0.0638	0.286	0.6	0.38	58	bat wing area (forewing only)
	0.04	0.286	0.6	0.38	93	Maniraptoran wing area (both wings)
	0.0718	0.286	0.6	0.38	52	bat wing area (both wings)
	0.032	0.286	0.6	0.45	138	Maniraptoran wing area (forewing only)
	0.0638	0.286	0.6	0.45	69	bat wing area (forewing only)
	0.04	0.286	0.6	0.45	110	Maniraptoran wing area (both wings)
	0.0718	0.286	0.6	0.45	61	bat wing area (both wings)
	0.032	0.286	0.6	0.7	214	Maniraptoran wing area (forewing only)
	0.0638	0.286	0.6	0.7	108	bat wing area (forewing only)
	0.04	0.286	0.6	0.7	172	Maniraptoran wing area area (both wings)
	0.0718	0.286	0.6	0.7	96	bat wing area (both wings)
Ambopteryx IVPP V24192	0.0132	0.185	0.385	0.23	171	Maniraptoran wing area area (forewing only)
	0.0263	0.185	0.385	0.23	86	bat wing area (forewing only)
	0.0165	0.185	0.385	0.23	137	Maniraptoran wing area (both wings)
	0.029	0.185	0.385	0.23	78	bat wing area (both wings)
	0.0132	0.185	0.385	0.31	228	Maniraptoran wing area (forewing only)
	0.0263	0.185	0.385	0.31	114	bat wing area (forewing only)
	0.0165	0.185	0.385	0.31	182	Maniraptoran wing area (both wings)
	0.029	0.185	0.385	0.31	101	bat wing area (both wings)
	0.0132	0.185	0.385	0.38	282	Maniraptoran wing area (forewing only)
	0.0263	0.185	0.385	0.38	142	bat wing area (forewing only)
	0.0165	0.185	0.385	0.38	226	Maniraptoran wing area (both wings)
	0.029	0.185	0.385	0.38	128	bat wing area (both wings)
Microraptor gui BMNHC PH881	0.04	0.263	0.55	0.18	44	Mass based on FL
	0.04	0.263	0.55	0.24	59	Mass based on FC
Microraptor gui IVPP V13352	0.089	0.45	0.94	0.95	105	Based on [11]
	0.0899	0.41	0.863	0.5	55	Based on [12]
	0.05	0.269	0.58	0.2	41	Based on [13]

Archaeopteryx Berlin specimen						

B)

Taxon	Wing area (m^2)	Wing length (m)	Span (m)	Mass (kg)	Wing loading (Nm^{-2})	Notes
Yi qi STM 31-2	0.067	0.286	0.6	0.38	56	Pterosaur model
	0.067	0.286	0.6	0.45	66	Pterosaur model
	0.067	0.286	0.6	0.7	102	Pterosaur model
	0.025	0.286	0.6	0.38	149	Frog model
	0.025	0.286	0.6	0.45	176	Frog model
	0.025	0.286	0.6	0.7	274	Frog model
Ambopteryx IVPP V24192	0.029	0.185	0.385	0.23	78	Pterosaur model
	0.029	0.185	0.385	0.31	105	Pterosaur model
	0.029	0.185	0.385	0.38	128	Pterosaur model
	0.011	0.185	0.385	0.23	205	Frog model
	0.011	0.185	0.385	0.31	276	Frog model
	0.011	0.185	0.385	0.38	339	Frog model

Supporting Table 3. Related to Figure 6.

Mass and wing loading from extinct and extant gliders. Note we separate *Coelurosauravus* from *Weigeltisaurus* based on Bulanov & Sennikov 2015.

Genus	Species	Mass (g)	Wing loading (Nm^{-2})	Extant or extinct	Reference
Acrobates	*pygmaeus*	-	84-92	Extant	Stafford, B. J., R.W., T. & Kawamichi, T. Gliding behavior of Japanese giant flying squirrels (Petaurista Leucogenys). *Journal of Mammalogy* **82**, 553-562 (2002).
Aeromys	*tephromelas*	1,300	80	Extant	Thorington jr, R. W. & Heaney, L. R. Body proportions and gliding adaptations of flying squirrels (Petauristinae). *Journal of Mammalogy* **62**, 101-114 (1981).
Anomalurus	*derbianus*	-	69-93	Extant	Stafford, B. J., R.W., T. & Kawamichi, T. Gliding behavior of Japanese giant flying squirrels (Petaurista Leucogenys). *Journal of Mammalogy* **82**, 553-562 (2002).
Anomalurus	*peli*	-	103-139	Extant	Stafford, B. J., R.W., T. & Kawamichi, T. Gliding behavior of Japanese giant flying squirrels (Petaurista Leucogenys). *Journal of Mammalogy* **82**, 553-562 (2002).
Cephalotes	*atratus*	0.05	14	Extant	Socha, J. J., Jafari, F., Munk, Y. & Byrnes, G. How animals glide: from trajectory to morphology. *Canadian Journal of Zoology* **93**, 901-924 (2015).
Chrysopelea	*paradisi*	40.5	29	Extant	Socha, J. J., Jafari, F., Munk, Y. & Byrnes, G. How animals glide: from trajectory to morphology. *Canadian Journal of Zoology* **93**, 901-924 (2015).
Cynocephalus	*volans*	1500	38-48	Extant	Stafford, B. J., R.W., T. & Kawamichi, T. Gliding behavior of Japanese giant flying squirrels (Petaurista Leucogenys). *Journal of Mammalogy* **82**, 553-562 (2002).
Cypsilurus	hiraii	-	56	Extant	Hertel, H. *Take-off and flight of the flying fish.* 218-224 (Reinhold, 1966).
Cheilopogon	*cyanopterus*	300	55	Extant	Hertel, H. *Take-off and flight of the flying fish.* 218-224 (Reinhold, 1966).
Draco	*fimbriatus*	18.7	23.5	Extant	McGuire, J. A. & Dudley, R. The cost of living large: comparative gliding performance in flying lizards (Agamidae: *Draco*). *American Naturalist* **166**, 93-106 (2005).
Draco	*formosus*	8.8	14.1	Extant	McGuire, J. A. & Dudley, R. The cost of living large: comparative gliding performance in flying lizards (Agamidae: *Draco*). *American Naturalist* **166**, 93-106 (2005).
Draco	*haematopogon*	5.7	12.4	Extant	McGuire, J. A. & Dudley, R. The cost of living large: comparative gliding performance in flying lizards (Agamidae: *Draco*). *American Naturalist* **166**, 93-106 (2005).
Draco	*maculatus*	4	12.9	Extant	McGuire, J. A. & Dudley, R. The cost of living large: comparative gliding performance in flying lizards (Agamidae: *Draco*). *American Naturalist* **166**, 93-106 (2005).
Draco	*maximus*	15.6	16	Extant	McGuire, J. A. & Dudley, R. The cost of living large: comparative gliding performance in flying lizards (Agamidae: *Draco*). *American Naturalist* **166**, 93-106 (2005).
Draco	*melanopogon*	3.8	9.2	Extant	McGuire, J. A. & Dudley, R. The cost of living large: comparative gliding performance in flying lizards (Agamidae: *Draco*). *American Naturalist* **166**, 93-106 (2005).
Draco	*obscurus*	9.1	15.7	Extant	McGuire, J. A. & Dudley, R. The cost of living large: comparative gliding performance in flying lizards (Agamidae: *Draco*). *American Naturalist* **166**, 93-106 (2005).
Draco	*quinquefasciatus*	6.5	10.5	Extant	McGuire, J. A. & Dudley, R. The cost of living large: comparative gliding performance in flying lizards (Agamidae: *Draco*). *American Naturalist* **166**, 93-106 (2005).
Draco	*sumatranus*	6.2	14.8	Extant	McGuire, J. A. & Dudley, R. The cost of living large: comparative gliding performance in flying lizards (Agamidae: *Draco*). *American Naturalist* **166**, 93-106 (2005).
Draco	*taeniopterus*	3.3	10	Extant	McGuire, J. A. & Dudley, R. The cost of living large: comparative gliding performance in flying lizards (Agamidae: *Draco*). *American Naturalist* **166**, 93-106 (2005).
Draco	*blanfordi*	11.3	14.1	Extant	McGuire, J. A. & Dudley, R. The cost of living large: comparative gliding performance in flying lizards (Agamidae: *Draco*). *American Naturalist* **166**, 93-106 (2005).

Eoglaucomys	*fimbriatus*	-	88-95	Extant	Stafford, B. J., R.W., T. & Kawamichi, T. Gliding behavior of Japanese giant flying squirrels (Petaurista Leucogenys). *Journal of Mammalogy* **82**, 553-562 (2002).
Exocoetid	sp.	32.4	23	Extant	Socha, J. J., Jafari, F., Munk, Y. & Byrnes, G. How animals glide: from trajectory to morphology. *Canadian Journal of Zoology* **93**, 901-924 (2015).
Galeopterus	*variegatus*	1750	49-71	Extant	Stafford, B. J., R.W., T. & Kawamichi, T. Gliding behavior of Japanese giant flying squirrels (Petaurista Leucogenys). *Journal of Mammalogy* **82**, 553-562 (2002).
Glaucomys	*sabrinus*	-	56-61	Extant	Stafford, B. J., R.W., T. & Kawamichi, T. Gliding behavior of Japanese giant flying squirrels (Petaurista Leucogenys). *Journal of Mammalogy* **82**, 553-562 (2002).
Glaucomys	*volans*	70	50	Extant	Thorington jr, R. W. & Heaney, L. R. Body proportions and gliding adaptations of flying squirrels (Petauristinae). *Journal of Mammalogy* **62**, 101-114 (1981).
Glaucomys	*sabrinus*	140	50	Extant	Thorington jr, R. W. & Heaney, L. R. Body proportions and gliding adaptations of flying squirrels (Petauristinae). *Journal of Mammalogy* **62**, 101-114 (1981).
Hylopetes	*platyurus*	41	40	Extant	Thorington jr, R. W. & Heaney, L. R. Body proportions and gliding adaptations of flying squirrels (Petauristinae). *Journal of Mammalogy* **62**, 101-114 (1981).
Hylopetes	*spadiceus*	63	50	Extant	Thorington jr, R. W. & Heaney, L. R. Body proportions and gliding adaptations of flying squirrels (Petauristinae). *Journal of Mammalogy* **62**, 101-114 (1981).
Petaurillus	*kinlochii*	20	40	Extant	Thorington jr, R. W. & Heaney, L. R. Body proportions and gliding adaptations of flying squirrels (Petauristinae). *Journal of Mammalogy* **62**, 101-114 (1981).
Petaurista	*elegans*	-	110	Extant	Stafford, B. J., R.W., T. & Kawamichi, T. Gliding behavior of Japanese giant flying squirrels (Petaurista Leucogenys). *Journal of Mammalogy* **82**, 553-562 (2002).
Petaurista	*petaurista*	-	93	Extant	Stafford, B. J., R.W., T. & Kawamichi, T. Gliding behavior of Japanese giant flying squirrels (Petaurista Leucogenys). *Journal of Mammalogy* **82**, 553-562 (2002).
Petaurista	*petaurista*	-	80	Extant	Stafford, B. J., R.W., T. & Kawamichi, T. Gliding behavior of Japanese giant flying squirrels (Petaurista Leucogenys). *Journal of Mammalogy* **82**, 553-562 (2002).
Petaurista	*petaurista*	-	104	Extant	Stafford, B. J., R.W., T. & Kawamichi, T. Gliding behavior of Japanese giant flying squirrels (Petaurista Leucogenys). *Journal of Mammalogy* **82**, 553-562 (2002).
Petaurista	*petaurista*	-	120	Extant	Stafford, B. J., R.W., T. & Kawamichi, T. Gliding behavior of Japanese giant flying squirrels (Petaurista Leucogenys). *Journal of Mammalogy* **82**, 553-562 (2002).
Petaurista	sp.	-	86-109	Extant	Stafford, B. J., R.W., T. & Kawamichi, T. Gliding behavior of Japanese giant flying squirrels (Petaurista Leucogenys). *Journal of Mammalogy* **82**, 553-562 (2002).
Petaurista	*elegans*	920	110	Extant	Thorington jr, R. W. & Heaney, L. R. Body proportions and gliding adaptations of flying squirrels (Petauristinae). *Journal of Mammalogy* **62**, 101-114 (1981).
Petaurista	*breviceps*	73.4	30.3	Extant	Bishop, K. L. Aerodynamic force generation, performance and control of body orientation during gliding in sugar gliders (*Petaurus breviceps*). *The Journal of Experimental Biology* **210**, 2593-2606 (2007).
Petauroides	*volans*	-	134-143	Extant	Stafford, B. J., R.W., T. & Kawamichi, T. Gliding behavior of Japanese giant flying squirrels (Petaurista Leucogenys). *Journal of Mammalogy* **82**, 553-562 (2002).
Petaurus	*australis*	-	63-81	Extant	Stafford, B. J., R.W., T. & Kawamichi, T. Gliding behavior of Japanese giant flying squirrels (Petaurista Leucogenys). *Journal of Mammalogy* **82**, 553-562 (2002).
Petaurus	*breviceps*	-	45-59	Extant	Stafford, B. J., R.W., T. & Kawamichi, T. Gliding behavior of Japanese giant flying squirrels (Petaurista Leucogenys). *Journal of Mammalogy* **82**, 553-562 (2002).
Petinomys	*vordemanni*	36	30	Extant	Thorington jr, R. W. & Heaney, L. R. Body proportions and gliding adaptations of flying squirrels (Petauristinae). *Journal of Mammalogy* **62**, 101-114 (1981).
Petinomys	*setosus*	39	40	Extant	Thorington jr, R. W. & Heaney, L. R. Body proportions and gliding adaptations of flying squirrels (Petauristinae). *Journal of Mammalogy* **62**, 101-114 (1981).

Petinomys	*genibarbis*	108	50	Extant	Thorington jr, R. W. & Heaney, L. R. Body proportions and gliding adaptations of flying squirrels (Petauristinae). *Journal of Mammalogy* **62**, 101-114 (1981).
Pteromyscus	*pulverulentu s*	266	40	Extant	Thorington jr, R. W. & Heaney, L. R. Body proportions and gliding adaptations of flying squirrels (Petauristinae). *Journal of Mammalogy* **62**, 101-114 (1981).
Ptychozoon	*kuhli*	11.1	31	Extant	Young, B. A., Lee, C. E. & Daley, K. M. On a flap and a foot: aerial locomotion in the "flying" gecko, *Pychozoon kuhli*. *Journal of Herptology* **36**, 412-418 (2002).
Sthenoteuthi s	*pteropus*	4	11.5	Extant	Socha, J. J., Jafari, F., Munk, Y. & Byrnes, G. How animals glide: from trajectory to morphology. *Canadian Journal of Zoology* **93**, 901-924 (2015).
Weigeltisaur us	*Jaekeli*	270	102	Extinct	Evans, S. E. The gliding reptiles of the Upper Permian. *Zoological Journal of the Linnean Society* **76**, 97-123 (1982).
Kuehneosuc hus	*latissimus*	0.4-0.56	97-135	Extinct	Stein, K., Palmer, C., Gill, P. G. & Benton, M. J. The aerodynamics of the British late Triassic Kuehneosauridae. *Palaeontology* **51**, 967-981 (2008).
Coelurosaur avus	*elivensis*	-	107.9	Extinct	McGuire, J. A. & Dudley, R. The biology of gliding in flying lizards (Genus *Draco*) and their Fossil and Extant Analogs. *Integrative and Comparative Biology* **51**, 983-990 (2011).
Icarosaurus	*seifkeri*	11.2	6.3	Extinct	McGuire, J. A. & Dudley, R. The biology of gliding in flying lizards (Genus *Draco*) and their Fossil and Extant Analogs. *Integrative and Comparative Biology* **51**, 983-990 (2011).
Xianglong	*zhaoi*	3.95	23.5	Extinct	Li, P. P., Gao, K., Hou, L. H. & Xu, X. A gliding lizard from the Early Cretaceous of China. *Proceedings of the National Academy of Sciences* **104**, 5507-5509 (2007).
Kuehneosau rus	*latus*	-	156.9	Extinct	McGuire, J. A. & Dudley, R. The biology of gliding in flying lizards (Genus *Draco*) and their Fossil and Extant Analogs. *Integrative and Comparative Biology* **51**, 983-990 (2011).

Supporting Table 4. Related to Figure 5 and main text.

A) Specific lift values generated during take-off using an estimated total flight muscles mass of 10% total body mass (this includes all muscles utilized during the flight stroke, not simply the pectoralis major). Values in bold indicate flight was possible.

B) Specific lift values generated during take-off using an estimated total flight muscles mass of 8% total body mass (this includes all muscles utilized during the flight stroke, not simply the pectoralis major). Values in bold indicate flight was possible.

A)

Taxon	Wing length (m)	Span (m)	Mass (kg)	Notes	FMR	Power output 225 WKg^{-1}	Power output 250 WKg^{-1}	Power output 287 Wkg^{-1}
Yi qi STM 31-2	0.286	0.6	0.38		0.04	9.69	**10.76**	**12.36**
	0.286	0.6	0.45		0.05	8.99	**9.99**	**11.47**
	0.286	0.6	0.7		0.07	7.40	8.23	9.45
Ambopteryx IVPP V24192	0.185	0.385	0.23		0.02	8.31	9.23	**10.59**
	0.185	0.385	0.31		0.03	7.33	8.14	9.34
	0.185	0.385	0.38		0.04	6.64	7.37	8.46
Microraptor gui BMNHC PH881	0.263	0.55	0.18	Mass based on FL	0.02	**12.51**	**13.89**	**15.95**
	0.263	0.55	0.24	Mass based on FC	0.02	**11.02**	**12.24**	**14.05**
Microraptor gui IVPP V13352	0.45	0.94	0.95	Based on [11]	0.10	9.46	**10.51**	**12.07**
	0.41	0.863	0.5	Based on [12]	0.05	**11.67**	**12.97**	**14.89**
Archaeopteryx Berlin specimen	0.269	0.58	0.2	Based on [13]	0.02	**12.49**	**13.87**	**15.93**

B)

Taxon	Wing length (m)	Span (m)	Mass (kg)	Notes	FMR	Power output 225 WKg^{-1}	Power output 250 WKg^{-1}	Power output 287 Wkg^{-1}
Yi qi STM 31-2	0.286	0.6	0.38		0.03	8.55	9.50	**10.91**
	0.286	0.6	0.45		0.04	7.94	8.82	**10.12**
	0.286	0.6	0.7		0.06	6.53	7.26	8.34
Ambopteryx IVPP V24192	0.185	0.385	0.23		0.02	6.46	7.18	8.25
	0.185	0.385	0.31		0.02	5.86	6.51	7.47
	0.185	0.385	0.38		0.03	7.33	8.14	9.35
Microraptor gui BMNHC PH881	0.263	0.55	0.18	Mass based on FL	0.01	**11.04**	**12.26**	**14.08**
	0.263	0.55	0.24	Mass based on FC	0.02	9.72	**10.80**	**12.40**
Microraptor gui IVPP V13352	0.45	0.94	0.95	Based on [11]	0.08	8.35	9.28	**10.65**
	0.41	0.863	0.5	Based on [12]	0.04	**11.67**	**12.97**	**14.89**
Archaeopteryx Berlin specimen	0.269	0.58	0.2	Based on [13]	0.02	**12.49**	**13.87**	**15.93**

Supporting Table 6. **Related to Transparent methods.**
Body weight support values from flap running. Values greater than 100% body weight support are denoted by a bolded 1+ and indicating take-off achieved. A) Coefficient of lift (CL)=2, B) CL=1.5, C) CL=1

A)

					**Body weight support**										
**OTU**	**Wing area model**	**Flapping (hz)**	**Mass (kg)**	**Flap angle (radians)**	**t=0**	**t=1**	**t=2**	**t=3**	**t=4**	**t=5**	**t=6**	**t=7**	**t=8**	**t=9**	**t=10**
Yi	Bird	10	0.38	0.87	0	0.02	0.04	0.06	0.09	0.13	0.19	0.28	0.41	0.59	0.84
		10	0.45	0.87	0	0.01	0.02	0.04	0.05	0.07	0.1	0.14	0.19	0.25	0.34
		9	0.7	0.87	0	0	0.01	0.01	0.01	0.02	0.02	0.03	0.03	0.04	0.05
		4	0.38	0.87	0	0	0	0	0.01	0.01	0.01	0.01	0.01	0.01	0.02
		4	0.45	0.87	0	0	0	0	0	0	0.01	0.01	0.01	0.01	0.01
		3	0.7	0.87	0	0	0	0	0	0	0	0	0	0	0
	Bat	10	0.38	0.87	0.01	0.07	0.17	0.36	0.71	**1+**	**1+**	**1+**	**1+**	**1+**	**1+**
		10	0.45	0.87	0	0.05	0.1	0.19	0.34	0.6	**1+**	**1+**	**1+**	**1+**	**1+**
		9	0.7	0.87	0	0.01	0.03	0.05	0.07	0.1	0.14	0.2	0.28	0.4	0.56
		4	0.38	0.87	0	0.01	0.01	0.02	0.02	0.03	0.04	0.06	0.08	0.11	0.15
		4	0.45	0.87	0	0	0.01	0.01	0.01	0.02	0.02	0.03	0.04	0.05	0.06
		3	0.7	0.87	0	0	0	0	0	0	0.01	0.01	0.01	0.01	0.01
Ambopteryx	Bird	11	0.31	0.87	0	0	0	0	0.01	0.01	0.01	0.01	0.01	0.01	0.01
		4	0.31	0.87	0	0	0	0.01	0.02	0.03	0.03	0.04	0.07	0.07	0.09
	Bat	11	0.31	0.87	0	0	0	0	0	0	0.01	0.01	0.01	0.01	0.01
		4	0.31	0.87	0	0	0	0	0	0	0	0	0	0	0.01
Microraptor	BMNH C PH881	9	0.18	0.87	0.01	0.07	0.16	0.34	0.7	**1+**	**1+**	**1+**	**1+**	**1+**	**1+**
		10	0.24	0.87	0.01	0.05	0.11	0.22	0.41	0.76	**1+**	**1+**	**1+**	**1+**	**1+**
	IVPP V13352	11	0.95	0.87	0.01	0.1	0.23	0.47	0.9	**1+**	**1+**	**1+**	**1+**	**1+**	**1+**
		12	0.5	0.87	0.01	0.41	**1+**	**1+**	**1+**	**1+**	**1+**	**1+**	**1+**	**1+**	**1+**
Archaeopteryx		13	0.2	0.87	0.01	0.15	0.45	**1+**	**1+**	**1+**	**1+**	**1+**	**1+**	**1+**	**1+**
Microraptor	BMNH C PH881	9	0.18	1.22	0.01	0.18	0.53	**1+**	**1+**	**1+**	**1+**	**1+**	**1+**	**1+**	**1+**
		10	0.24	1.22	0.01	0.13	0.36	0.83	**1+**	**1+**	**1+**	**1+**	**1+**	**1+**	**1+**
	IVPP V13352	11	0.95	1.22	0.01	0.28	0.76	**1+**	**1+**	**1+**	**1+**	**1+**	**1+**	**1+**	**1+**
		12	0.5	1.22	0.02	**1+**	**1+**	**1+**	**1+**	**1+**	**1+**	**1+**	**1+**	**1+**	**1+**
Archaeopteryx		13	0.2	1.22	0.01	0.45	**1+**	**1+**	**1+**	**1+**	**1+**	**1+**	**1+**	**1+**	**1+**

B)

					**Body weight support**										
**OTU**	**Wing area model**	**Flapping (hz)**	**Mass (kg)**	**Flap angle (radians)**	**t=0**	**t=1**	**t=2**	**t=3**	**t=4**	**t=5**	**t=6**	**t=7**	**t=8**	**t=9**	**t=10**
Yi	Bird	10	0.38	0.87	0	0.01	0.02	0.03	0.04	0.06	0.08	0.11	0.15	0.2	0.26
		10	0.45	0.87	0	0.01	0.01	0.02	0.03	0.04	0.05	0.06	0.08	0.1	0.12
		9	0.7	0.87	0	0	0	0.01	0.01	0.01	0.01	0.01	0.02	0.02	0.02
		4	0.38	0.87	0	0	0	0	0	0	0	0.01	0.01	0.01	0.01
		4	0.45	0.87	0	0	0	0	0	0	0	0	0	0	0
		3	0.7	0.87	0	0	0	0	0	0	0	0	0	0	0
	Bat	10	0.38	0.87	0	0.04	0.09	0.16	0.28	0.47	0.8	**1+**	**1+**	**1+**	**1+**
		10	0.45	0.87	0	0.03	0.05	0.09	0.15	0.23	0.36	0.55	0.85	**1+**	**1+**
		9	0.7	0.87	0	0.01	0.02	0.02	0.03	0.05	0.06	0.08	0.11	0.14	0.18
		4	0.38	0.87	0	0	0.01	0.01	0.01	0.02	0.02	0.03	0.03	0.04	0.05
		4	0.45	0.87	0	0	0	0.01	0.01	0.01	0.01	0.01	0.02	0.02	0.03
		3	0.7	0.87	0	0	0	0	0	0	0	0	0	0	0.01
Ambopteryx	Bird	11	0.31	0.87	0	0	0	0	0	0	0	0.01	0.01	0.01	0.01
		4	0.31	0.87	0	0	0.01	0.01	0.01	0.01	0.02	0.02	0.03	0.03	0.04
	Bat	11	0.31	0.87	0	0	0	0	0	0	0	0.01	0	0	0.01
		4	0.31	0.87	0	0	0	0	0	0	0	0.03	0	0	0
Microraptor	BMNH C PH881	9	0.18	0.87	0	0.04	0.08	0.15	0.27	0.46	0.81	**1+**	**1+**	**1+**	**1+**
		10	0.24	0.87	0	0.03	0.06	0.1	0.17	0.28	0.44	0.71	**1+**	**1+**	**1+**
	IVPP V13352	11	0.95	0.87	0	0.05	0.12	0.21	0.36	0.6	0.99	**1+**	**1+**	**1+**	**1+**
		12	0.5	0.87	0.01	0.21	0.6	**1+**	**1+**	**1+**	**1+**	**1+**	**1+**	**1+**	**1+**
Archaeopteryx		13	0.2	0.87	0.01	0.08	0.21	0.45	0.93	**1+**	**1+**	**1+**	**1+**	**1+**	**1+**
Microraptor	BMNH C PH881	9	0.18	1.22	0.01	0.1	0.25	0.54	**1+**	**1+**	**1+**	**1+**	**1+**	**1+**	**1+**
		10	0.24	1.22	0.01	0.07	0.17	0.35	0.66	**1+**	**1+**	**1+**	**1+**	**1+**	**1+**
	IVPP V13352	11	0.95	1.22	0.01	0.15	0.36	0.75	**1+**	**1+**	**1+**	**1+**	**1+**	**1+**	**1+**
		12	0.5	1.22	0.01	0.63	**1+**	**1+**	**1+**	**1+**	**1+**	**1+**	**1+**	**1+**	**1+**
Archaeopteryx		13	0.2	1.22	0.01	0.23	0.7	**1+**	**1+**	**1+**	**1+**	**1+**	**1+**	**1+**	**1+**

C)

					**Body weight support**										
**OTU**	**Wing area model**	**Flapping (hz)**	**Mass (kg)**	**Flap angle (radians)**	**t=0**	**t=1**	**t=2**	**t=3**	**t=4**	**t=5**	**t=6**	**t=7**	**t=8**	**t=9**	**t=10**
Yi	Maniraptoran	10	0.38	0.87	0	0.01	0.01	0.01	0.02	0.02	0.03	0.04	0.05	0.06	0.07
		10	0.45	0.87	0	0	0.01	0.01	0.01	0.01	0.02	0.02	0.03	0.03	0.04
		9	0.7	0.87	0	0	0	0	0	0	0.01	0.01	0.01	0.01	0.01
		4	0.38	0.87	0	0	0	0	0	0	0	0	0	0	0
		4	0.45	0.87	0	0	0	0	0	0	0	0	0	0	0
		3	0.7	0.87	0	0	0	0	0	0	0	0	0	0	0
	Bat	10	0.38	0.87	0	0.02	0.04	0.06	0.09	0.13	0.19	0.28	0.4	0.58	0.83
		10	0.45	0.87	0	0.01	0.02	0.04	0.05	0.07	0.1	0.14	0.19	0.25	0.34
		9	0.7	0.87	0	0	0.01	0.01	0.01	0.02	0.02	0.03	0.03	0.04	0.05
		4	0.38	0.87	0	0	0	0	0.01	0.01	0.01	0.01	0.01	0.01	0.02
		4	0.45	0.87	0	0	0	0	0	0	0.01	0.01	0.01	0.01	0.01
		3	0.7	0.87	0	0	0	0	0	0	0	0	0	0	0
Ambopteryx	Maniraptoran	11	0.31	0.87	0	0	0	0	0	0	0	0	0	0	0
		4	0.31	0.87	0	0	0	0	0	0.01	0.01	0.01	0.01	0.01	0.01
	Bat	11	0.31	0.87	0	0	0	0	0	0	0	0	0	0	0
		4	0.31	0.87	0	0	0	0	0	0	0	0	0	0	0
Microraptor	BMNHC PH881	9	0.18	0.87	0	0.02	0.03	0.05	0.08	0.13	0.19	0.27	0.4	0.59	0.86
		10	0.24	0.87	0	0.01	0.03	0.04	0.06	0.08	0.12	0.16	0.23	0.31	0.43
	IVPP V13352	11	0.95	0.87	0	0.02	0.05	0.08	0.12	0.18	0.25	0.36	0.5	0.71	0.99
		12	0.5	0.87	0.01	0.09	0.21	0.42	0.79	**1+**	**1+**	**1+**	**1+**	**1+**	**1+**
Archaeopteryx		13	0.2	0.87	0	0.04	0.08	0.14	0.24	0.41	0.68	**1+**	**1+**	**1+**	**1+**
Microraptor	BMNHC PH881	9	0.18	1.22	0	0.04	0.09	0.17	0.29	0.5	0.84	**1+**	**1+**	**1+**	**1+**
		10	0.24	1.22	0	0.03	0.07	0.12	0.19	0.3	0.47	0.74	**1+**	**1+**	**1+**
	IVPP V13352	11	0.95	1.22	0	0.07	0.14	0.25	0.41	0.66	**1+**	**1+**	**1+**	**1+**	**1+**
		12	0.5	1.22	0.01	0.25	0.69	**1+**	**1+**	**1+**	**1+**	**1+**	**1+**	**1+**	**1+**
Archaeopteryx		13	0.2	1.22	0.01	0.1	0.24	0.5	**1+**	**1+**	**1+**	**1+**	**1+**	**1+**	**1+**

References

Amador, L. I., Simmons, N. B. & Giannini, N. P. Aerodynamic reconstruction of the primitive fossil bat *Onychonycteris finneyi* (Mammalia: Chiroptera). *Biology Letters* **15**, 20180857 (2019).

Bulanov, V. V. & Sennikov, A. G. New data on the morphology of the Late Permian gliding reptile *Coelurosauravus elivensis* Piveteau. *Paleontological Journal* **49**, 413-423 (2015).

Greenewalt, C. H. The flight of birds: the significant dimensions, their departure from the requirements for dimensional similarity, and the effect on flight aerodynamics of that departure. *Transactions of the American Philosophical Society* **65(4)**: 1-67 (1975).

Xu, X. *et al.* A bizarre Jurassic maniraptoran theropod with preserved evidence of membranous wings. *Nature* **521**, 70-73 (2015).

Wang, M., O'Connor, J. K., Xu, X. & Zhou, Z. A new Jurassic scansoriopterygid and the loss of membranous wings in theropod dinosaurs. *Nature* **569**, 256-259 (2019).
